# Discovery of Bacterial Unspecific Peroxygenases

**DOI:** 10.1021/acs.biochem.6c00230

**Published:** 2026-06-30

**Authors:** Esteban Lopez-Tavera, Anton A. Stepnov, Nikolai S. Ersdal, Marta Barros-Reguera, Ronja Marlonsdotter Sandholm, Sabina Leanti La Rosa, Morten Sørlie, Vincent G. H. Eijsink, Gustav Vaaje-Kolstad

**Affiliations:** Faculty of Chemistry, Biotechnology and Food Science, 56625 Norwegian University of Life Sciences (NMBU), P.O. Box 5003, Ås N-1432, Norway

## Abstract

Unspecific peroxygenases (UPOs, EC 1.11.2.1) are promising biocatalysts for the oxyfunctionalization of organic molecules and the synthesis of industrially relevant compounds because of their vast repertoire of catalyzed reactions. To date, thousands of putative UPO genes have been identified in eukaryotic genomes, most of them in the Ascomycota and Basidiomycota phyla, and several UPOs have been characterized. Remarkably, no related enzymes have been reported in prokaryotic organisms. Here, we describe the discovery of a diverse family of bacterial heme-thiolate peroxygenases through structure database mining, followed by functional characterization of selected representatives. The bacterial proteins, termed bacterial UPOs (BUPOs), display clear structural homology to family I (short) fungal UPOs despite low sequence identity (<20%). Expression of one of these proteins (*Hyd*BUPO) in its native host (*Hydrogenophaga* *sp*. A37) was confirmed by proteomics. Several BUPOs were cloned and expressed in *Escherichia coli*. In biochemical assays, the BUPOs were able to catalyze one-electron oxidation (peroxidase activity) of ABTS and 2,6-dimethoxyphenol, as well as two-electron oxidation (peroxygenase activity) of naphthalene, indole, 3-phenyl-1-propanol, and 16-hydroxypalmitic acid, using hydrogen peroxide as a cosubstrate. These enzymes thus represent a previously unknown group of bacterial heme-thiolate peroxygenases that share key structural and functional features with eukaryotic UPOs, offering potential candidates for the development of industrially relevant biocatalysts.

## Graphical abstract

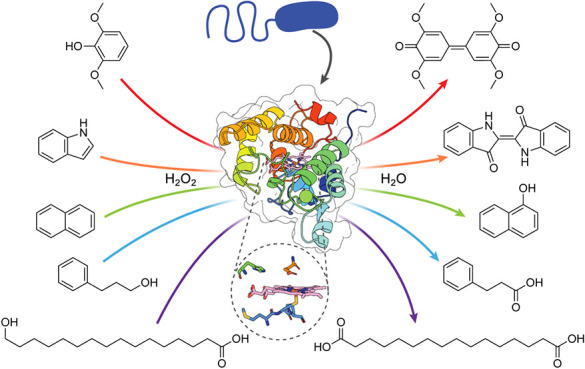

## Introduction

Since their discovery in 2004,^1^ unspecific peroxygenases (UPOs, EC 1.11.2.1) have been shown to catalyze a remarkably large set of oxyfunctionalization reactions in a broad spectrum of substrates.^2,3^ Among the UPO-catalyzed reactions are the oxidation of aryl alcohols to aldehydes and carboxylic acids;^1^ hydroxylation of aromatic substrates such as toluene and naphthalene;^4^ oxidative cleavage of ether bonds;^5^ oxidation of saturated alkanes, aliphatic alcohols, and fatty acids to alcohols, ketones, and carboxylic acids;^6–8^ and the epoxidation of unsaturated compounds.^9^ Unlike cytochrome P450 monooxygenases (CYP450), which are capable of similar oxyfunctionalization reactions, UPOs do not require cofactors such as NAD­(P)H or flavin-containing redox partners for catalysis but depend solely on hydrogen peroxide as a cosubstrate.^1^ The vast repertoire of peroxygenation reactions catalyzed by UPOs, together with the simple reaction setups requiring only H_2_O_2_, make this enzyme family attractive for industrial applications. However, despite recent advances in optimizing UPO production,^10–12^ low expression yields still represent a major bottleneck for the use of UPOs in industrial applications.^2,13^

When UPOs were first described, no sequence homology was found with any previously known peroxidases or cytochrome P450 peroxygenases, and the only known relative was a heme chloroperoxidase from the filamentous fungus *Caldariomyces fumago* (*Cfu*CPO).^14^ Since then, mining of genomic databases has revealed thousands of putative UPO genes in several fungal phyla (mainly Ascomycota and Basidiomycota) and Oomycota,^13^ classified in two large families: I, “short” (29 kDa in average), or II, “long” (44 kDa in average),^15^ several of which have been studied and shown to encode enzymes with a wide diversity of substrates, catalytic activities, and stereoselectivities.^16,17^ For over 20 years, these enzymes have been considered exclusive to fungi and Oomycota, with no confirmed representation in other eukaryotes or any prokaryotes.

Owing to the development of the powerful structure-based search engine FoldSeek^18^ and the availability of vast databases of accurate protein structure models such as the AlphaFold database (over 200 million proteins),^19,20^ it is now possible to go beyond sequence similarity when searching for functionally related proteins. Furthermore, in the case of enzymes that depend on cofactors, like heme in UPOs, the availability of tools such as AlphaFold3 ^21^ for accurate prediction of cofactor binding greatly enhances the capabilities of in silico mining of novel proteins.

Here, we report the discovery of a new family of bacterial heme-thiolate enzymes, termed bacterial unspecific peroxygenases (BUPOs), characterized by a thiolate-coordinated heme cofactor and a tertiary structure resembling that of eukaryotic UPOs. We verified the expression of one of these enzymes in its native organism, and then successfully produced several BUPOs in *Escherichia coli* and demonstrated their peroxygenase activity. These findings significantly expand the known redox enzyme repertoire of prokaryotes and reveal potential candidates for the development of industrial biocatalysts.

## Materials and Methods

### Structure-Based Search with FoldSeek and Identification of the BUPO Dataset

Two short-type fungal UPOs, *Mro*UPO (PDB 7zbp) and *Hsp*UPO (PDB 7o1r), and one family II (“long”) member, *Aae*UPO’s evolved mutant PaDa-I (PDB 5OXU), were used as templates to search for bacterial homologues using FoldSeek.^18^ The search was performed against the AlphaFold database AFDB50, using 3Di/AA mode (local alignment using the 3Di alphabet + BLOSUM62, as described in^18^) and a taxonomic filter restricting the results to bacteria (eubacteria). Only hits with a probability greater than 0.5 and sequences longer than 150 amino acids were considered positive hits. The resulting amino acid sequences (19 proteins in total) were searched in UniProt BLAST individually to expand the dataset; identified proteins with E-values lower than 10^–18^ and longer than 150 AA were added to the original set, and duplicate sequences were removed using Cd-hit^22^ with a 100% identity cutoff. SignalP 6.0 ^23^ was used to predict the presence of secretion signals, and InterPro Scan^24^ was used to annotate protein domains.

To reduce the redundancy of the dataset prior to further analyses of the sequences, Cd-hit was used again, this time with an identity cutoff of 90%, resulting in a reduced set of 91 sequences.

### Phylogenetic Analysis

To explore the relatedness of the putative bacterial UPOs and fungal UPOs, it was necessary to remove the additional domains of multidomain proteins; thus, the AlphaFold models of two bacterial proteins containing a C-terminal cytochrome C peroxidase domain were visually analyzed to determine the limit of the BUPO domain. These two sequences were manually truncated and aligned with the reduced dataset of bacterial proteins using Clustal Omega.^25^ This initial multiple sequence alignment (MSA) was truncated at the limit of the BUPO domain, and a new MSA was made, now including a set of 25 previously studied fungal UPOs (Table S1), using the online MAFFT server.^26^

The MSA was used to calculate the pairwise sequence identity percentage using the following equation:

$$\%\;\mathrm{identity}=\frac{\mathrm{number of identical non‐gap residue pairs}}{\mathrm{length of the shortest sequence in the pair}}\times 100$$

Key regions of the alignment were used to make sequence logos using the online WebLogo 3 server.^27^ Before the phylogenetic analysis, the 6 most divergent bacterial sequences were removed from the alignment, and then a neighbor-joining phylogenetic tree was built in the MAFFT server^26^ with the alignment containing a total of 110 sequences—85 bacterial and 25 fungal—using the JTT substitution model and 100 bootstrap resampling rounds. The tree was annotated with the online tool iTol,^28^ coupling it with the output from InterProScan and SignalP as described above.

By analyzing the phylogenetic tree and the predicted structures, 13 putative bacterial UPOs were selected for expression in *E. coli*, aiming to cover sufficient diversity to increase the chances of successful expression.

### Structure Prediction and Analysis

Structure predictions of the 85 BUPOs included in the phylogenetic analysis were obtained using the AlphaFold3 server,^21^ including a heme b cofactor and a Mg^2+^ ion as ligands. The predicted structures were aligned to the crystal structure of *Hsp*UPO (PDB 7o1r) using the TM-align algorithm in the Python package tmtools, version 0.2.0,^29–31^ from which the TM-scores relative to *Hsp*UPO were obtained.

### Materials and Expression Vectors

All chemicals were purchased from Sigma-Aldrich (St. Louis, MO, USA), unless otherwise stated. Palmitic acid was purchased from Fluka Chemie (Buchs, Switzerland), and 5-aminolevulinic acid was purchased from Chem-Impex (Wood Dale, IL, USA). The *Hydrogenophaga* *sp.* A37 isolate^32^ was kindly provided by Prof. Åsa Frostegård at the Norwegian University of Life Sciences.

Genes encoding putative bacterial UPOs were codon-optimized for expression in *E. coli*, using the GenSmart codon optimization tool (https://www.genscript.com/tools/gensmart-codon-optimization), with added 6-His tags either at the N-terminus or the C-terminus, as chosen by inspecting their AlphaFold models to avoid adding tags at termini that were either buried or close to the active site tunnel. For the proteins with a predicted signal peptide, three versions were designed: native (with the native signal peptide), truncated (removed signal peptide), and with a PelB signal peptide (PelB-SP). In the truncated and pelB-SP variants of predicted lipoproteins, the lipidation-site cysteine was also excluded. The amino acid sequences of the expressed proteins are shown in Table S2. The constructs were synthesized and cloned into the pET-29b­(+) vector (*Nde*I/*Xho*I restriction sites) by Twist Bioscience (South San Francisco, CA, USA) or GenScript (Rijswijk, Netherlands) (detailed in Table S2).

### Resequencing of the *Hydrogenophaga* sp. A37’s Genome, Assembly, and Annotation

*Hydrogenophaga* *sp.* A37 was cultured in 1/10 diluted TSB medium (Tryptic Soy Broth, Merck, Darmstadt, Germany) for 48 h at 22 °C with shaking at 200 rpm. Genomic DNA was extracted using the DNeasy PowerSoil Pro kit (QIAGEN), following the manufacturer’s instructions. DNA concentration was measured using a NanoDrop One spectrophotometer and a Qubit 3.0 fluorometer with the dsDNA High Sensitivity assay kit (Thermo Fisher Scientific). DNA quality was assessed by gel electrophoresis on a Bio-Rad Gel Doc EZ Imager. As this genome was sequenced with other samples, a sequencing library was prepared using the Native Barcoding kit SQK-NBD114.24 (Oxford Nanopore Technologies), following the manufacturer’s protocols. The library was loaded onto two FLO-MIN114 R10.4 flow cells and sequenced for 48 h on a MinION device using MinKNOW v4.0.5. POD5 files were base-called and demultiplexed with Dorado v0.5.0 (https://github.com/nanoporetech/dorado) using the superaccurate model (dna_r10.4.1_e8.2_400bps_sup@v4.3.0). Raw reads were quality-filtered with FiltLong v0.2.1 (https://github.com/rrwick/Filtlong) using the parameters --min_length 3000, --keep_percent 90, and assembled using Flye v2.9.2 (--nano_hq, --min-overlap 1500, default settings).^33^ Contigs were initially polished with two consecutive rounds using Medaka v2.0.1 (-m r1041_e82_400bps_sup_v5.0.0; https://github.com/nanoporetech/medaka), followed by mapping raw reads to the consensus sequence generated by Medaka using minimap2 v2.28-r1209.^34^ The alignment was polished using Racon v1.5.0 (-m 8 -x −6 -g −8 -w 500) (https://github.com/isovic/racon). Assembly completeness was evaluated with CheckM.^35^ GTDB-Tk with GTDB release 220 was used to determine the taxonomic assignment.^36^ Functional annotations were obtained using the NCBI Prokaryotic Genome Annotation Pipeline (PGAP) v6.10.^37^

### Proteomic Analysis of *Hydrogenophaga* *sp.* A37

*Hydrogenophaga* *sp.* A37 was grown in quadruplicate in 1/10 TSB for 48 h at 22 °C with shaking at 200 rpm. Samples (5 mL) were harvested at the mid-exponential growth phase (OD_600_ 0.6). Cells were separated from the supernatant by centrifugation (6,000 × g, 20 min, 4 °C), resuspended in 50 mM Tris-HCl pH 7.5, 100 mM NaCl, 0.1% (v/v) Triton X-100, 1 mM dithiothreitol (DTT), and disrupted by bead-beating (three 60 s cycles) with a FastPrep24 (MP Biomedicals, CA) at 6.5 m s^–1^. After removal of cell debris by centrifugation (16,000 × g, 10 min, 4 °C), the proteins in the supernatant were precipitated by adding 50% ice-cold TCA with a 1:4 ratio and incubated at 4 °C overnight. Precipitated proteins were collected by centrifugation (15,000 × g, 15 min, 4 °C) and washed by adding 300 μL ice-cold wash buffer followed by centrifugation (15,000 × g, 15 min, 4 °C). The wash buffer was decanted before air-drying the proteins. Proteins were resuspended in 46 μL lysis buffer (5% SDS and 50 mM triethylammonium bicarbonate pH 8.5). Protein digestion was performed using S-Trap Mini Columns (Protifi, Fairport, NY, USA) according to the manufacturer’s instructions, with 20 mM DTT for reduction and 40 mM iodoacetamide for alkylation. The peptides were dried in a speed-vac and then dissolved in 1% (v/v) formic acid. Samples were injected into an Ultimate 3000 nano ultrahigh-performance liquid chromatography system (Dionex, Sunnyvale, CA, USA) connected to a Q-Exactive quadrupole-orbitrap mass spectrometer (Thermo Scientific, Bremen, Germany) equipped with a nanoelectrospray ion source. Chromatographic separation was performed on a nanoViper Acclaim PepMap 100 C18 column (3 μm, 100 Å, 50 cm; Dionex, Sunnyvale, CA, USA) at a flow rate of 300 nL min^–1^. Peptides were eluted using a gradient from 12% to 43% solvent B over 93 min, followed by an increase to 90% B in 6 min (solvent A: 0.1% formic acid in water; solvent B: 80% acetonitrile, 0.08% formic acid). The mass spectrometer operated in data-dependent mode, alternating between Orbitrap MS (R = 70,000) and HCD Orbitrap MS/MS (R = 35,000). The AGC target was set to 1,000,000 charges with a maximum injection time of 128 ms. The 10 most intense ions were selected for fragmentation per cycle, with a 20 s dynamic exclusion for previously fragmented precursors.

MS raw files were processed using FragPipe v21.1 (https://fragpipe.nesvilab.org/), with MSFragger v4.0,^38^ IonQuant v1.10.12,^39^ and Philosopher v5.1.0 ^40^ for protein identification and label-free quantification (LFQ). MS and MS/MS spectra were searched against the complete *Hydrogenophaga* *sp.* A37 proteome (5,226 proteins, derived from the newly assembled genome), supplemented with common contaminants (e.g., keratins, trypsin, and bovine serum albumin) and a decoy database of reversed sequences to estimate false discovery rates (FDRs). The final protein library comprised 10,688 proteins. Trypsin was set as the proteolytic enzyme, with one missed cleavage allowed. Carbamidomethylation of cysteines was set as a fixed modification, while variable modifications included methionine oxidation and pyro-glutamate formation at N-terminal glutamines. Protein identifications were filtered to achieve a 1% FDR. A protein was considered “present” if detected in at least three of the four biological replicates.

### Screening Enzyme Candidates for Activity

*E. coli* BL21 Star (DE3) competent cells (Thermo Fisher Scientific, Waltham, USA) were transformed with the plasmids, and transformants were selected on lysogeny broth (LB) agar plates containing 50 μg mL^–1^ kanamycin and 5 mg mL^–1^ glucose to minimize basal expression of T7 polymerase. Liquid cultures were prepared in sterile 12-well plates with 1.5 mL LB with 50 μg mL^–1^ kanamycin and 5 mg mL^–1^ glucose per well. Each well was inoculated with a single colony, and the plates were incubated overnight in thermomixers (Eppendorf, Hamburg, Germany) at 37 °C and 300 rpm. The resulting cultures were mixed 7:3 with sterile 86% (w/w) glycerol in a polypropylene 96-well plate, sealed, and stored at −80 °C until further use.

Expression tests were carried out using sterile 1.3 mL 96-deep-well plates and ZYP-5052 autoinduction medium,^41^ consisting of 5 mg mL^–1^ yeast extract, 10 mg mL^–1^ tryptone, 50 mM Na_2_HPO_4_, 50 mM NaH_2_PO_4_, 25 mM NH_4_SO_4_, 5 mg mL^–1^ glycerol, 0.5 mg mL^–1^ glucose, 2 mg mL^–1^ lactose, and 2 mM MgSO_4_, with 200 μg mL^–1^ kanamycin for selection, and 50 μM FeCl_3_ and 500 μM 5-aminolevulinic acid to promote heme biosynthesis. The glycerol stocks were thawed, and 3 μL of each clone was used to inoculate 600 μL of medium. For each clone, two wells were inoculated, and two wells inoculated with nontransformed *E. coli* BL21 Star (DE3) were included, using the same medium but without kanamycin. The plate was sealed with a breathable sterile rayon film (Nunc, Thermo Fisher; Rochester, NY, USA) and incubated at 30 °C for 24 h in a thermomixer set to 800 rpm. The cultures were then transferred into 1.5 mL microcentrifuge tubes. The duplicate samples corresponding to the same experimental conditions (i.e., the same enzyme variants or the same control cultures) were pooled together, resulting in a 1.2 mL final volume. Next, the cells were pelleted by centrifugation (10 min at 14,000 × g), and the pellets were stored at −20 °C until further use.

Cell lysis was performed by resuspending the cells in 250 μL of BugBuster protein extraction reagent (Merck, Darmstadt, Germany) containing 0.4 mg mL^–1^ lysozyme (Roche Diagnostics, Mannheim, Germany). The cell suspensions were incubated in a thermomixer at 25 °C and 300 rpm for 30 min, followed by centrifugation (20 min at 20,000 × g). This initial screen was designed as a simple, uniform expression comparison of multiple BUPO constructs rather than an optimization of secretion or subcellular localization; therefore, total soluble extracts were used for all constructs, regardless of signal peptide presence, to maximize recovery and comparability. Proteins were purified from the supernatants using Pierce High-Capacity Ni-IMAC magnetic beads (Thermo Fisher Scientific, Waltham, MA, USA). Twenty microliters of a 25% (w/w) bead suspension was loaded into 1.5 mL microcentrifuge tubes and equilibrated with 500 μL of 50 mM sodium phosphate buffer, pH 7.4, containing 5 mM imidazole and 300 mM NaCl. The tubes were placed on a magnetic rack, and the supernatants were replaced with 750 μL of the same equilibration buffer. 250 μL of clarified lysates was added into each tube, followed by incubation for 10 min at room temperature in a rotary shaker (Multi RS-60; BioSan, Riga, Latvia) set to 25 rpm. The supernatants were removed using the magnetic rack, and the beads were washed two times with 500 μL of 50 mM sodium phosphate buffer, pH 7.4, containing 10 mM imidazole and 300 mM NaCl. The proteins were eluted with 200 μL of 50 mM sodium phosphate buffer, pH 7.4, supplied with 500 mM imidazole and 300 mM NaCl.

To probe enzymatic activity, 10 μL of the resulting protein samples was transferred into 96-well microtiter plates and mixed with 80 μL of a 1 mM 2,2’-azino-bis­(3-ethylbenzothiazoline-6-sulfonic acid) (ABTS) solution in 50 mM sodium phosphate buffer, pH 6.0. The reactions were started by adding 10 μL of a 10 mM H_2_O_2_ solution in Milli-Q water, followed by mixing for 30 s at 600 rpm using a Varioskan LUX plate reader (Thermo Fisher Scientific, Waltham, MA, USA). The final concentrations of ABTS and H_2_O_2_ in the reaction mixtures were 0.8 mM and 1 mM, respectively. The experiments were carried out for 1 h at 25 °C. Product formation (i.e., the generation of ABTS radicals) was followed by measuring the optical absorbance at 418 nm.

### Production and Purification of BUPOs

The glycerol stocks of the strains expressing *Age*BUPO (native variant), *Kal*BUPO (pelB-SP variant), and *Hyd*BUPO (native variant) were used to start 20 mL precultures in 100 mL flasks containing LB medium supplemented with 50 μg mL^–1^ kanamycin and 5 mg mL^–1^ glucose. The precultures were incubated overnight at 37 °C and 200 rpm in an Ecotron incubator (Infors HT, Bottmingen, Switzerland). Ten mL of the overnight precultures was used to inoculate shake flasks containing 1 L of ZYP-5052 autoinduction medium, supplemented with 500 μM δ-aminolevulinic acid, followed by incubation at 200 rpm and 30 °C for 24 h (*Age*BUPO and *Hyd*BUPO) or 16 °C for 96 h (*Kal*BUPO) in an Ecotron incubator. Afterward, the cultures were harvested by centrifuging at 8,000 × g for 10 min. The pellets from bacteria expressing *Age*BUPO and *Hyd*BUPO were resuspended in metal affinity chromatography binding buffer (20 mM sodium phosphate, 0.5 M NaCl, 40 mM imidazole, pH 7.4) and lysed by sonicating with a VibraCell ultrasonic disintegrator equipped with a microtip probe (Sonics, Newtown, CT, USA) for 10 min at 28% amplitude, pulsing 5 s on and 5 s off. The lysates were clarified by centrifugation for 10 min at 20,000 × g at 4 °C. The clarified lysates were manually loaded with a syringe onto a 5 mL HisTrap FF Crude column (Cytiva, Marlborough, MA, USA), pre-equilibrated with binding buffer. The columns were then washed with 10 column volumes of binding buffer, after which the proteins were eluted with 5 column volumes of elution buffer (20 mM sodium phosphate, 0.5 M NaCl, 500 mM imidazole, pH 7.4).

After the expression of *Kal*BUPO with the pelB signal peptide, the clarified culture medium was visibly red and showed increased viscosity, suggesting partial cell lysis during growth. The clarified medium was therefore used directly as the starting material for purification by loading it (1 L) onto a pre-equilibrated 5 mL HisTrap FF Crude column (Cytiva) using a BioLogic LP System (Bio-Rad, Hercules, CA, USA). We note that the red color of the medium alone does not distinguish between the heme-loaded protein and free heme. The column was then washed with 10 column volumes of binding buffer, and the UPO was eluted with 10 column volumes of elution buffer.

The proteins were concentrated to reduce their volumes to <2 mL using Amicon Ultra-15 centrifugal filters (Merck KGaA, Darmstadt, Germany) with a molecular weight cutoff of 10 kDa and filtered through 0.22 μm filters to remove potential protein precipitates. Each protein was then further purified by size-exclusion chromatography using a ProteoSEC Dynamic 16/60 3–70 HR column (Protein Ark, Sheffield, UK), operated at a 1 mL min^–1^ flow rate and equilibrated with 20 mM sodium phosphate buffer, pH 7.0, containing 200 mM NaCl. The fractions showing absorbance at 420 nm were pooled and concentrated as described above, and the heme-containing protein concentration was estimated by measuring the absorbance at 420 nm (reflecting the presence of UPO-bound heme) using the extinction coefficient of *Mro*UPO (ε_420_ = 115 mM^–1^ cm^–1^),^42^ assuming similar extinction coefficients for the BUPOs. The extinction coefficients were subsequently assessed by iron quantification using ICP-MS, confirming that they were close to that of *Mro*UPO (see below in the section “Quantification of iron and magnesium with ICP-MS”).

### Production and Purification of *Hsp*UPO

The gene encoding the fungal UPO from *Hypoxylon* *sp., Hsp*UPO (UniProt accession A0A1Y2TH07), was codon-optimized for expression in *Komagataella phaffii* and synthesized by Twist Bioscience (South San Francisco, CA, USA), replacing the native signal peptide sequence by that of the alpha factor signal peptide, and including 30 bp overhangs on each end, homologous to the expression vector pBSY3Z (Bisy GmbH, Hofstaetten a. d. Raab, Austria) for Gibson assembly cloning. The vector was linearized by PCR using the primers 5′-TTTAATTGTAAGTCTTGACTAGAGCAAGTG-3′ and 5′-GCGGCCGCTCAAGAGGAT-3′ and the Q5 High-Fidelity polymerase (New England Biolabs, USA), treated with the restriction enzyme *Dpn*I (New England Biolabs, USA), and purified with the DNA Clean-up and Concentration kit (Zymo Research, USA). The *Hsp*UPO gene fragment was then assembled into the expression vector by using a NEBuilder HiFi DNA Assembly kit (New England Biolabs, USA) according to the manufacturer’s instructions. The assembled DNA was introduced into *E. coli* TOP10 cells (Invitrogen, Thermo Fisher Scientific, USA), and transformants were selected by plating on LB agar plates containing 25 μg mL^–1^ of Zeocin (Gibco, Thermo Fisher Scientific, USA). Single colonies were cultured overnight in 5 mL LB containing 25 μg mL^–1^ of Zeocin for plasmid propagation. Plasmid DNA was then purified with the EZNA Plasmid DNA Mini Kit I (Omega Bio-Tek, USA) and sequenced by Eurofins Genomics (Ebersberg, Germany) using Sanger sequencing. A version of the gene with an added affinity tag, StrepTagII (WSHPQFEK), at the N-terminus of the UPO downstream of the signal peptide was produced by amplifying the sequence-verified plasmid with the primers 5′-TTTTTCGAACTGCGGGTGAGACCA-AGCTTCGGCCTCTCTCTTCTCG-3′ and 5′-CTTGGTCTCACCCGCAGTTCGAAAAAGCTCCAT-CTCCATCTTCTGGTTG-3′, following the same procedure for cloning, selection, and sequence verification as described above. Sequence-verified plasmids were then linearized with *Swa*I (New England Biolabs, USA) and used to transform electrocompetent *K. phaffii* MutS BSYBG11 cells (Bisy GmbH, Austria). Successful transformants were selected on YPD agar plates containing 100 μg mL^–1^ Zeocin. Single colonies were used to inoculate 5 mL cultures in BMD1 medium (200 mM potassium phosphate buffer, pH 6.0, 13.4 g L^–1^ yeast nitrogen base, 0.4 mg L^–1^ biotin, 10 g L^–1^ glucose), which were incubated at 30 °C and 200 rpm for 66 h. After that, 0.5 mL of BMM10 medium (200 mM potassium phosphate buffer, pH 6.0, 13.4 g L^–1^ yeast nitrogen base, 0.4 mg L^–1^ biotin, 5% (v/v) methanol) was added for induction, with subsequent additions of 50 μL of pure methanol at 74, 90, and 98 h. Cultures were harvested at 114 h by centrifugation (5,000 × g, 10 min, at 4 °C). The supernatants were used to carry out an activity assay with 2,6-DMP (2,6-dimethoxyphenol, Merck, Germany), as described below in the section “Peroxidase colorimetric assay with 2,6-DMP” and the clone with the highest activity was used to produce the enzyme at a larger scale. Then, *Hsp*UPO and *Hsp*UPO-StrepTagII were produced in 2-L baffled Erlenmeyer flasks containing 500 mL of BMD1 medium. The cultures were incubated at 30 °C and 200 rpm for 66 h, after which 50 mL of BMM10 medium was added for induction, with subsequent additions of 2.5 mL of pure methanol every 12 h. At 160 h, the culture medium was harvested by centrifugation at 8,000 × g and 4 °C for 20 min. The cell pellet was discarded, and the supernatant was filtered through a 0.22 μm filter (SteriTop, Merck, Rahway, NJ, USA) and concentrated 10-fold using a Tangential Flow Filtration (TFF) cassette (Vivaflow, Sartorius, Göttingen, Germany).

*Hsp*UPO (with no affinity tag) was purified from the concentrated culture supernatant using ion exchange chromatography (HiTrap Capto Q, 5 mL; Cytiva, Marlborough, MA, USA), with the column pre-equilibrated in 20 mM Tris-HCl buffer, pH 8.0, and proteins eluted with a 0–0.5 M NaCl gradient over 40 column volumes. *Hsp*UPO-StrepTagII was purified by loading the concentrated culture supernatant onto a 5 mL StrepTrap XT affinity column (Cytiva, Marlborough, MA, USA), pre-equilibrated in 100 mM Tris-HCl, 150 mM NaCl, 1 mM EDTA, pH 8, and proteins were eluted using 3 column volumes of a buffer with the same composition but containing 50 mM biotin. Fractions with the highest absorbance at 420 nm were pooled, concentrated, and buffer-exchanged into 20 mM sodium phosphate, pH 7.0, using an Amicon Ultra-15 centrifugal filter (Merck KGaA, Darmstadt, Germany) with a molecular weight cutoff of 10 kDa. The protein concentration was estimated by measuring absorbance at 420 nm, using the extinction coefficient of *Mro*UPO (115 mM^–1^ cm^–1^),^42^ assuming a similar value for *Hsp*UPO. The extinction coefficient was subsequently assessed by iron quantification through ICP-MS (see below, in section “Quantification of iron and magnesium with ICP-MS”).

### UV–vis Spectra

The UV–vis spectra of *Age*BUPO, *Kal*BUPO and *Hyd*BUPO were recorded using a NanoPhotometer C40 (Implen GmbH, München, Germany). The enzyme preparations were diluted to approximately 10 μM (based on the absorbance at 420 nm) in 20 mM sodium phosphate buffer, pH 7.0, and the resting-state spectra were recorded in a quartz cuvette with a 1 cm path length. Then, a few grains of sodium dithionite were added to the cuvette, and the sample was gently mixed by inversion before measuring the UV–vis spectrum again, yielding the spectrum for the reduced enzyme. The reduced enzyme solution was then flushed with pure CO gas, and the CO-enzyme complex spectrum was measured.

### Quantification of Iron and Magnesium with ICP-MS

*Age*BUPO, *Kal*BUPO, *Hyd*BUPO, and *Hsp*UPO-StrepTagII were diluted in 20 mM sodium phosphate buffer (pH 7.0) to 1–2 μM heme-containing protein, using the concentration estimated with the absorbance at 420 nm as described above. Buffer alone was used to quantify the background Fe and Mg content, and deionized water was used as a blank for the analysis. One milliliter of each sample (in triplicate) was weighed in 15 mL centrifuge tubes, and 0.75 mL of ultrapure concentrated HNO_3_ was added. The samples were heated to 90 °C for 1 h and then diluted with deionized water to a final volume of 10 mL. The concentrations of Fe and Mg were quantified using a triple-quadrupole ICP-MS system (8900 ICP-QQQ, Agilent, Santa Clara, CA, USA) operated in He-KED mode at masses 56 and 24 amu for Fe and Mg, respectively, with indium as the internal standard. The limits of detection (3 × SD from blank samples, n = 5) and quantification (10 × SD from the blank samples, n = 5) were 0.0007 and 0.0023 mg kg^–1^ for Fe, and 0.0007 and 0.0022 mg kg^–1^ for Mg, respectively.

### Peroxidase Colorimetric Assay with ABTS

The reactions were carried out in 96-well plates, in a total volume of 200 μL, containing 0.8 mM ABTS and 0.5 μM enzyme in 100 mM sodium citrate buffer, pH 5.5. The reference enzyme, *Hsp*UPO, was used at 0.05 μM. Control reactions with 0.5 μM hemin chloride or buffer instead of enzyme were included. The experiment was started by dispensing 10 μL of 20 mM H_2_O_2_ (to a final concentration of 1 mM), or water in control reactions, using a Varioskan Lux plate reader (Thermo Fisher Scientific), and product formation was monitored by measuring the absorbance at 418 nm for 15 min, using the built-in path length correction method. The concentration of oxidized ABTS was calculated using the molar extinction coefficient ε_418_ = 36 mM^–1^ cm^–1^.^43^ In the experiment for determining the pH dependency, the same conditions were used, except 0.1 μM enzyme was used for all enzymes, and reactions were carried out in McIlvaine buffer (75 mM) with pH ranging from 3.0 to 7.6.

### Peroxidase Colorimetric Assay with 2,6-DMP

In the peroxidase reaction with 2,6-dimethoxyphenol (2,6-DMP), the formation of the dimerized product coerulignone was monitored following absorbance at 469 nm (ε_469_ = 27.5 mM^–1^ cm^–1^).^43^ The reaction mixture contained 2 mM 2,6-DMP, 2 mM H_2_O_2_, 100 mM potassium phosphate buffer (pH 6.0), and either 0.5 μM bacterial enzyme or 0.05 μM *Hsp*UPO. Control reactions with 5 μM hemin chloride, 5 μM FeCl_3_ or buffer instead of enzyme were included. Twenty miroliters of a 10x enzyme solution was added in a 96-well plate, and the experiment was started by dispensing 180 μL of a reaction mix containing the rest of the components using a Varioskan Lux plate reader (Thermo Fisher Scientific; Rochester, NY, USA). The absorbance at 469 nm was monitored for 15 min using the built-in path length correction method.

### Peroxygenase Colorimetric Assay with Indole

The peroxygenase reaction with indole, leading to the formation of indigo, was monitored by measuring absorbance at 670 nm (ε_670_ = 4.8 mM^–1^ cm^–1^).^43^ The reaction mixture contained 2 mM indole (diluted 10 times from a stock prepared in 50% (v/v) acetonitrile, leading to a final acetonitrile content of 5%), 2 mM H_2_O_2_, 100 mM potassium phosphate buffer (pH 7.0), and 5 μM bacterial enzyme or 0.5 μM *Hsp*UPO. Control reactions with 5 μM hemin chloride, 5 μM FeCl_3_ or buffer instead of enzyme were included. Twenty microliters of a 10x enzyme solution was added in a 96-well plate, and the experiment was started by dispensing 180 μL of a reaction mix containing the rest of the components using a Varioskan Lux plate reader (Thermo Fisher Scientific; Rochester, NY, USA). The absorbance at 670 nm was monitored for 15 min using the built-in path length correction method.

### Peroxygenase Spectrophotometric Assay with Naphthalene

In the peroxygenase reactions with naphthalene, the formation of 1-naphthol was monitored by measuring the absorbance at 324 nm. The reaction mixtures contained 1 mM naphthalene, 5 μM bacterial enzyme or 0.5 μM *Hsp*UPO, 100 mM sodium citrate buffer (pH 5.5), and 10% acetone. Control reactions with 5 μM hemin chloride, 5 μM FeCl_3_ or water instead of enzyme were included. The experiment was started by dispensing 10 μL of 20 mM H_2_O_2_ (to a final concentration of 1 mM) or buffer, using a Varioskan Lux plate reader (Thermo Fisher Scientific; Rochester, NY, USA), and the absorbance at 324 nm was monitored for 15 min. A standard curve of 1-naphthol (5–1000 μM) was used to calculate the concentration of product.

### Peroxygenase Spectrophotometric Assay with Veratryl Alcohol

The peroxygenase reaction with veratryl alcohol, leading to the formation of veratraldehyde, was monitored by measuring absorbance at 310 nm (ε_310_ = 9.3 mM^–1^ cm^–1^).^1^ The reaction mixture contained 5 mM veratryl alcohol, 1 mM H_2_O_2_, 100 mM potassium phosphate buffer (pH 7.0), and 5 μM bacterial enzyme or 0.5 μM *Hsp*UPO. Control reactions with 5 μM hemin chloride, 5 μM FeCl_3_ or buffer instead of enzyme were included. All components except H_2_O_2_ were added in a UV-transparent 96-well plate, and the experiment was started by dispensing 20 μL of 10 mM H_2_O_2_ (to a final concentration of 1 mM) using a Varioskan Lux plate reader (Thermo Fisher Scientific, Rochester, NY, USA). The absorbance at 310 nm was monitored for 15 min, using the built-in path length correction method.

### Monitoring the Enzymatic Oxidation of 3-Phenyl-1-propanol (3PP)

The oxidation of 3PP by BUPOs and *Hsp*UPO was assessed in 50 mM sodium phosphate buffer (pH 6.0), supplied with 1 mM substrate and 5 μM enzyme. The experiments were initiated by adding H_2_O_2_ to a 1 mM final concentration and were carried out at 30 °C (using a thermomixer; Eppendorf, Hamburg, Germany) or at room temperature. Reactions with hemin chloride substituting the enzyme and reactions with just H_2_O_2_ and the substrate were used as negative controls. For end-point experiments, the reaction samples were transferred to HPLC vials and immediately analyzed as described below. For time-course experiments, aliquots were taken at various time points, and the reactions were quenched by adding bovine liver catalase (Sigma-Aldrich, St. Louis, MO, USA) to a final concentration of 250 U mL^–1^. The reaction mixtures were analyzed by reverse-phase chromatography using a Dionex UltiMate 3000 RSLC system (Thermo Fisher Scientific, Waltham, MA, USA) equipped with a Zorbax RR Eclipse Plus C18 column (2.1 × 150 mm, 3.5 μm) and a Zorbax RRHD Eclipse Plus C18 guard column (2.1 × 5 mm, 1.8 μm) produced by Agilent (Santa Clara, CA, USA). Separation was carried out using 10 mM ammonium acetate (buffer A) and 100% acetonitrile (buffer B) at a 0.35 mL min^–1^ flow rate. The column was equilibrated for 5 min (99% buffer A, 1% buffer B), and then a linear gradient of buffer B (1–25%) was applied over 20 min at a column temperature of 30 °C. 3PP and its oxidized derivatives were detected by monitoring the UV absorbance at 260 nm.

### Oxidation of Palmitic Acid and Hydroxy-Palmitic Acid

Enzymatic reactions with palmitic acid and 16-hydroxypalmitic acid as substrates were carried out in 1.5 mL tubes, in a total volume of 100 μL, containing 5 μM of enzyme (except *Hsp*UPO, which was used at 0.5 μM) or hemin chloride, 0.1 mM substrate, 20% (v/v) acetone, and 10 mM ammonium acetate, pH 6.0. The experiments were started by adding H_2_O_2_ to a final concentration of 1 mM, followed by incubation at 30 °C and 1,000 rpm in a thermomixer (Eppendorf, Hamburg, Germany) for 1 h. Next, 200 μL of 90% (v/v) acetonitrile containing 6 mM ammonium acetate was added, and the reactions were stirred for 5 min at 1,000 rpm in the thermomixer at 20 °C to allow protein precipitation and favor the dissolution of the analytes, followed by centrifugation for 5 min at 20,000 × g to remove protein precipitates. 60 μL of the supernatant was transferred to HPLC vials, and the samples were analyzed by LC-CAD/MS using a Dionex UltiMate 3000 RSLC system (Thermo Fisher Scientific, Waltham, MA, USA) equipped with a Zorbax RR Eclipse Plus C18 column (2.1 × 150 mm, 3.5 μm) and a Zorbax RRHD Eclipse Plus C18 guard column (2.1 × 5 mm, 1.8 μm) produced by Agilent (Agilent, Santa Clara, CA, USA). The chromatography system was coupled, with a flow splitter 1:1, to a Corona Ultra charged aerosol detector (CAD) and a Velos Pro MS system (Thermo Fisher Scientific, Waltham, MA, USA). Chromatographic separation of the analytes was performed using a linear gradient from 50% to 99% eluent B (acetonitrile) over 2 min, followed by an isocratic hold at 99% B for 6 min, at a flow rate of 0.4 mL min^–1^. Eluent A was 10 mM ammonium acetate in water. The MS system was operated in negative ionization mode with a scanning range of 500–1,000 *m*/*z*. The CAD nebulizer temperature was set to 30 °C. 16-Hydroxyhexadecanoic acid and hexadecanedioic acid were used as standards to identify elution peaks and quantify the corresponding compounds by integrating the peak areas in the extracted ion chromatograms of their respective [M—H]^−^ *m*/*z* values (271.23 ± 0.2 and 285.21 ± 0.2). The amounts of keto and aldehyde products (isomeric) were estimated by integrating the peaks at *m*/*z* 269.21 ± 0.2 and calculating the concentrations using the standard curve of 16-hydroxypalmitic acid, assuming the same response factor due to the unavailability of authentic standards.

## Results

### Structure-Based Database Mining Reveals Bacterial UPOs

The development of high-precision structure prediction tools, such as AlphaFold,^19^ has revolutionized in silico enzyme discovery by allowing structure-based database searches rather than sequence-based searches. To identify putative BUPOs, two crystal structures of family I (“short”) fungal UPOs, *Mro*UPO (PDB 7ZBP) and *Hsp*UPO (PDB 7O1R), and one family II (“long”) member, *Aae*UPO’s evolved mutant PaDa-I (PDB 5OXU), were used as templates for searching similar structures using the FoldSeek online server,^18^ applying a taxonomic filter for bacteria (eubacteria). While no relevant hits were found for the long UPO, the search with short UPOs yielded a total of 21 bacterial proteins with high probability scores (>0.7) and sequence coverage (>70%, relative to the queried proteins). Despite the structural similarity, these proteins shared low sequence identity (<18%) with the queried fungal UPOs, which is most likely the reason why bacterial UPOs have remained undetected by sequence-based search algorithms.

Prediction of the structures of these proteins with a b-type heme cofactor, using the AlphaFold 3 server,^21^ showed a cysteine residue coordinating the heme in the same way as in fungal UPOs, adding to the notion that these proteins indeed are BUPOs. Two of the 21 proteins (UniProt accession numbers A0A4Q5XIQ3 and A0A2H9SVS9) had a histidine-coordinated heme and were excluded from further analyses.

To expand the dataset, the 19 bacterial sequences were searched in UniProt BLAST toward the UniProtKB database, which resulted in 169 additional proteins with sequence identities varying from 33% to 98% relative to the query sequences. The final set of putative bacterial UPOs consisted of 188 unique proteins (Supporting Dataset 1). Then, to decrease redundancy in subsequent analyses, the sequences were clustered using Cd-hit,^22^ keeping representative sequences with less than 90% sequence identity. This reduced set was used to build a multiple sequence alignment (MSA) with 25 previously studied fungal UPOs (Table S1) using the Mafft server.^26^

The final alignment, containing 85 bacterial and 25 fungal sequences, was used to build a neighbor-joining phylogenetic tree of UPOs and BUPOs (Figure 1), which shows that the BUPO sequences cluster separately from both long and short fungal UPOs. The tree reflects considerable diversity among the bacterial sequences. Clade I consists mostly of proteins with a lipoprotein signal peptide (Sec/SPII-type), according to predictions by the SignalP algorithm.^23^ In clade II, most proteins have a predicted Sec/SPI-type secretion signal peptide, indicating that they are exported from the cytoplasm. While most of the BUPO domains were not assigned to a protein family by InterPro Scan,^24^ the proteins in clade III could be assigned to the chloroperoxidase-like superfamily (ID IPR036851). Not surprisingly, clade III members are also the ones with the highest sequence identity (up to 19%) with fungal UPOs (Figure S1). Several clade III proteins have predicted N-terminal disordered regions. Proteins in clades IV and V have no obvious representative features, as recognized by InterPro. Finally, whereas clades I–V are single-domain proteins, clade VI has several members that consist of two domains: the N-terminal BUPO domain (observed in predicted structures; not identified by InterPro), followed by a transmembrane region and a C-terminal diheme cytochrome C peroxidase domain (InterPro code IPR047758).

**Figure 1:**
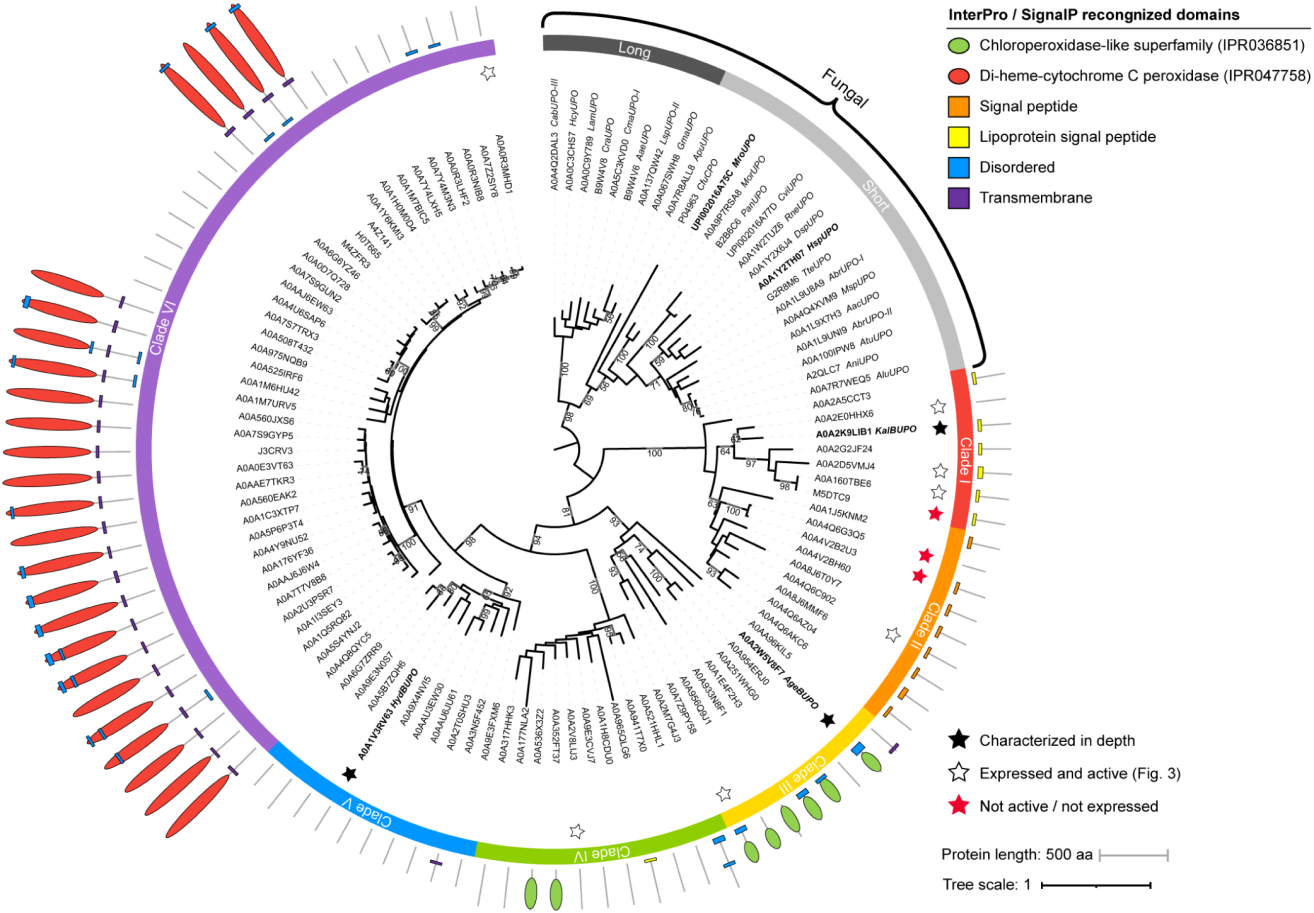
Phylogenetic tree of representative fungal and bacterial UPOs. The phylogenetic tree features 25 previously studied fungal UPOs (family I “short”, light gray; family II “long”, dark gray) and 85 putative bacterial UPOs, separated into 6 distinct clades (I–VI). The stars indicate sequences that were tested for expression in *E. coli* in this study. The black stars (★) indicate the proteins that were characterized in depth (see main text), the white stars (☆) indicate the proteins with activity observed in a rapid microtiter plate assay, and the red stars (★) indicate proteins with no detected activity/expression (Figure 3; see main text for details). Protein domains assigned using InterPro and signal peptides by SignalP are shown around the tree, with the N-terminus pointing toward the center of the tree, and the chain length scale (in number of amino acids) shown in gray. The tree scale (in number of amino acid substitutions per site) is shown in black. Bootstrap values ≥50 are shown next to the respective branches. The protein labeled A0A2A5CCT3 is not part of Clade I but is closely related and is predicted to be a lipoprotein, as are most of the Clade I members.

Analysis of regions containing key catalytic UPO motifs (Figure S2) revealed that the PCP motif, which includes the heme-coordinating cysteine (Cys39 in *Hsp*UPO), is conserved among the BUPOs, as well as an acid–base catalytic pair (Glu180 as acid–base catalyst and His110 as its charge stabilizer in *Hsp*UPO) that is known to participate in the heterolytic cleavage of H_2_O_2_.^13^ In some of the bacterial proteins, His110 is replaced by another basic residue (lysine or arginine). Of note, arginine is also observed in the acid–base pair in long UPOs.^13^ In contrast, residues Glu109, Asp111, and Ser113 in *Hsp*UPO, involved in the binding of a Mg^2+^ ion and highly conserved in fungal UPOs,^13^ seem to be completely absent in the bacterial proteins (Figure S2B). In fungal UPOs, the bound magnesium ion is thought to stabilize the strained porphyrin system of the heme cofactor.^13^

### The BUPOs Feature Key Structural Elements of Fungal UPOs

To couple the sequence analysis to structural information, we predicted the structures of the 85 putative BUPOs shown in Figure 1 using the AlphaFold 3 server,^21^ including a b-type heme cofactor and a Mg^2+^ ion as ligands. Like the fungal UPOs, BUPOs display a chloroperoxidase-like fold with an orthogonal bundle architecture of mainly alpha-helices. This architecture is exemplified by three BUPOs shown in Figure 2B–D, which correspond to the enzymes investigated in detail in this study (*Age*BUPO, *Kal*BUPO, and *Hyd*BUPO). The TM-scores obtained when comparing *Hsp*UPO (PDB 7o1r) with each of the three predicted BUPO structures shown in Figure 2B–D varied from 0.66 to 0.74, which is well above the cutoff (0.5) for defining structures as having mostly the same fold.^29–31^ The active site of UPOs is characterized by a cysteine coordinating the heme iron at the proximal side and an acid–base pair on the distal side of the heme that is necessary for the cleavage of hydrogen peroxide in the catalytic cycle.^44^ All of these elements are present in BUPOs (Figure 2F–H), and, as mentioned above, these residues are highly conserved among all putative bacterial UPOs (Figure S2). Note that the histidine in the conserved acid–base pair (His110 and Glu180 in *Hsp*UPO) is replaced by another basic residue (Lys95) in *Hyd*BUPO (Figure 2H). Notably, magnesium binding was predicted in a different loop for *Age*BUPO and *Kal*BUPO (Figure 2B,C) than in fungal UPOs. While the magnesium ion is predicted to bind in *Hyd*BUPO (Figure 2D,H) in an analogous location to that of fungal UPOs, later analysis by ICP-MS showed that none of these three bacterial proteins contained Mg^2+^ (see below).

**Figure 2:**
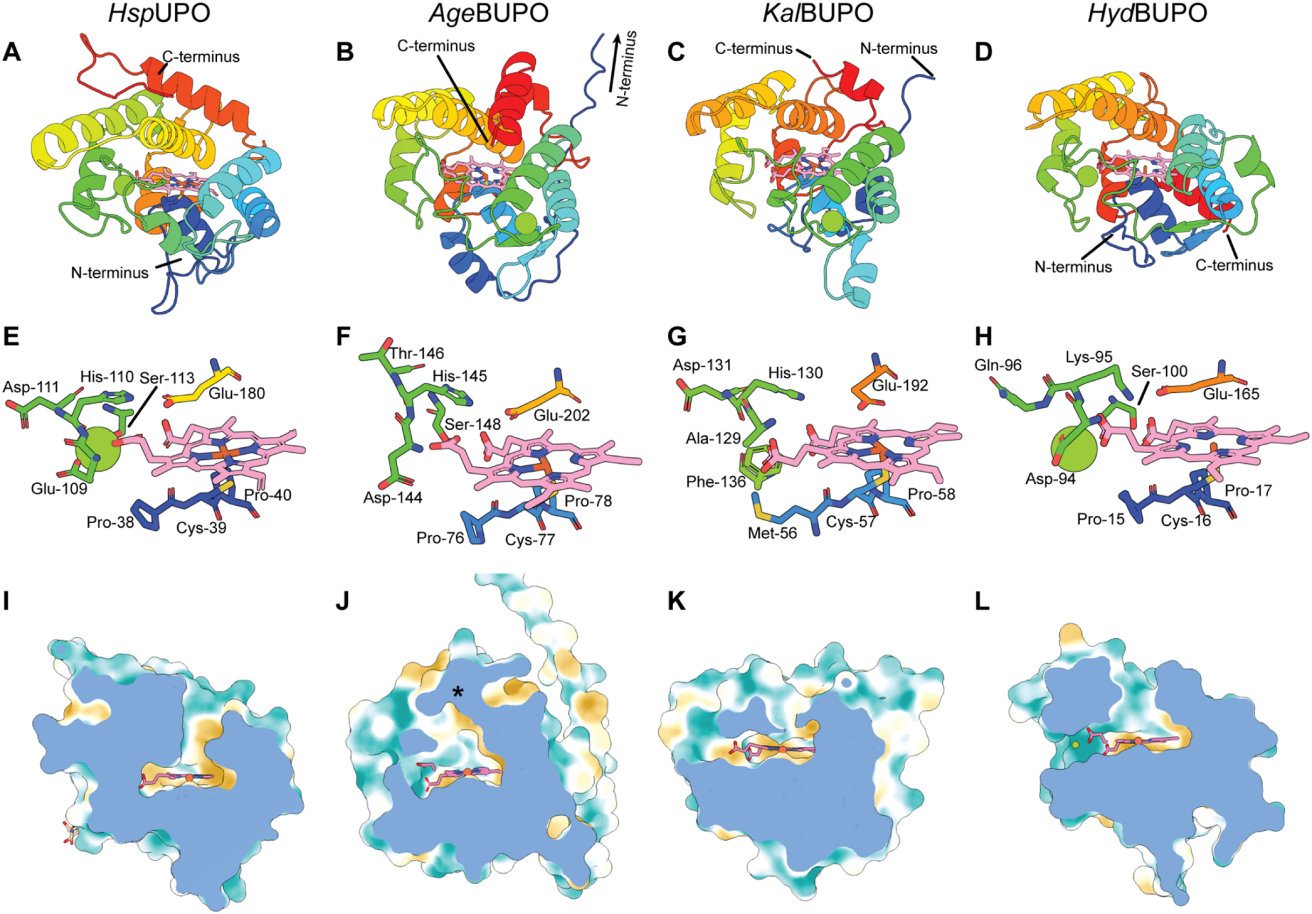
Structural features of bacterial UPOs and comparison with *Hsp*UPO. The short fungal UPO *Hsp*UPO (PDB 7o1r; A, E, I) is shown as a reference for comparison with the AlphaFold3-predicted structures of *Age*BUPO (B, F, J, clade 3), *Kal*BUPO (C, G, K, clade 1), and *Hyd*BUPO (D, H, L, clade 5). In the top row (A–D), the structures are colored by residue position to illustrate the topology and spatial arrangement of the α-helices. The UPO-defining conserved residues are shown in the second row (E–H), together with the heme group and van der Waals sphere of Mg^2+^ (green). The residue numbering is relative to the full peptide chain, including signal peptides. The solvent-excluded surface (I–L) is shown in a lipophilicity color scale, from gold (lipophilic) to teal (hydrophilic). The transversal view of the access tunnel (I–L) shows a second opening present in the BUPOs (extending to the left), where one of the heme propionate groups is exposed to the solvent. The C-terminus of *Age*BUPO shown in panels B and J (marked in J with a star symbol, *), as well as a long N-terminal region (truncated in the figure), have a very low prediction confidence (pLDDT value <50) and are likely to be misfolded in the model or inherently disordered. The AlphaFold3 models colored by pLDDT score are shown in Figure S4.

The predicted structures suggest diverse geometries and sizes for the active site access tunnels of the bacterial UPOs (Figure 2J–L). Whereas fungal UPOs only have one tunnel leading to the active site (e.g., *Hsp*UPO, Figure 2I), all BUPOs, except those in clade IV, are predicted to have a second tunnel leading to the heme cofactor, where one of the heme propionate groups is exposed to the solvent (Figure 2F–H and Figure S3). Notably, the location of the second tunnel entrance in BUPOs is similar to the location of the Mg^2+^ coordination site in fungal UPOs (Figure 2E–L).

### The Native Expression of a BUPO is Confirmed in *Hydrogenophaga* *sp.* A37 through Proteomics

Having identified BUPO candidates in sequence databases, we sought to experimentally confirm the presence of one such gene in its native organism’s genome and to determine whether the corresponding BUPO was natively expressed, in order to rule out the remote possibility that these bacterial genes represent sequencing artifacts or nonfunctional evolutionary remnants. For this purpose, we used *Hydrogenophaga* *sp*. A37, a denitrifying bacterium isolated from soil,^32^ which harbors the BUPO candidate with UniProt accession A0A1 V3RV63 (clade V), hereafter referred to as *Hyd*BUPO. At the start of this study, the genome assembly of this organism available in NCBI (GCA_002001205.1) had been generated using short-read Illumina sequencing. It comprised 195 contigs with an N50 of 56 kb and a total genome size of 5.6 Mb. Here, we generated an improved *Hydrogenophaga* sp. A37 genome assembly using Oxford Nanopore long-read sequencing (GCA_053010425.1). The new assembly consists of a single circular chromosome of 5,605,460 bp (assembly level: complete genome), with an overall G+C content of 65.5%. The genome achieved 80× coverage. Genome quality assessments indicated 99.1% completeness and 1.9% contamination. In total, 5295 CDS, 5226 genes, 45 tRNAs, and 3 complete rRNA were predicted. Sequence alignment using Minimap2 ^34^ and DGENIES^45^ revealed 99.86% identity with the earlier genome version, indicating high sequence identity and collinearity (Figure S5). With this improved genome, we generated a complete proteome to be used as the reference database for the proteomic analysis of *Hydrogenophaga* *sp*. A37. The bacterium was grown for 48 h at 22 °C in a diluted commercial medium (tryptone soy broth), and proteins were isolated from the cell pellet prior to trypsin digestion and LC-MS/MS analysis. A total of 2,179 proteins were detected (listed in Supporting Dataset 2), among which we identified *Hyd*BUPO (NCBI accession MGS5087929.1), thereby confirming that *Hyd*BUPO is a functional gene expressed in its native organism.

### Functional BUPOs Can Be Expressed in *Escherichia coli*

A set of 13 candidate BUPOs spanning the six different clades (Figure 1; named BU01-BU13) were selected for heterologous expression in *Escherichia coli*. For the 9 candidates with predicted signal peptides or N-terminal disordered regions, genes encoding three variants were synthesized: one with the native signal peptide, one with the pelB signal peptide, and a truncated variant with no signal peptide (see list in Table S2). For the remaining four candidates (BU5, BU7, BU12, and BU13) only the native form of the gene was synthesized. A microscale expression screen based on detecting activity toward 2,2′-azino-bis (3-ethylbenzothiazoline-6-sulfonic acid) (ABTS) showed that 10 of the 13 BUPO targets were successfully produced (Figure 3B). No activity was detected for BU07–BU09, and for several enzymes, only one or two of the expressed variants displayed measurable activity. The lack of activity (Figure 3B) in some candidate BUPOs was generally associated with the absence of detectable expression (Figure 3A), and thus it is unclear whether those enzymes would be active if expressed. In the case of BU01–3, the band observed around 65 kDa is likely an unrelated contaminant protein, and no band is observed at the expected size (23.8 kDa).

**Figure 3:**
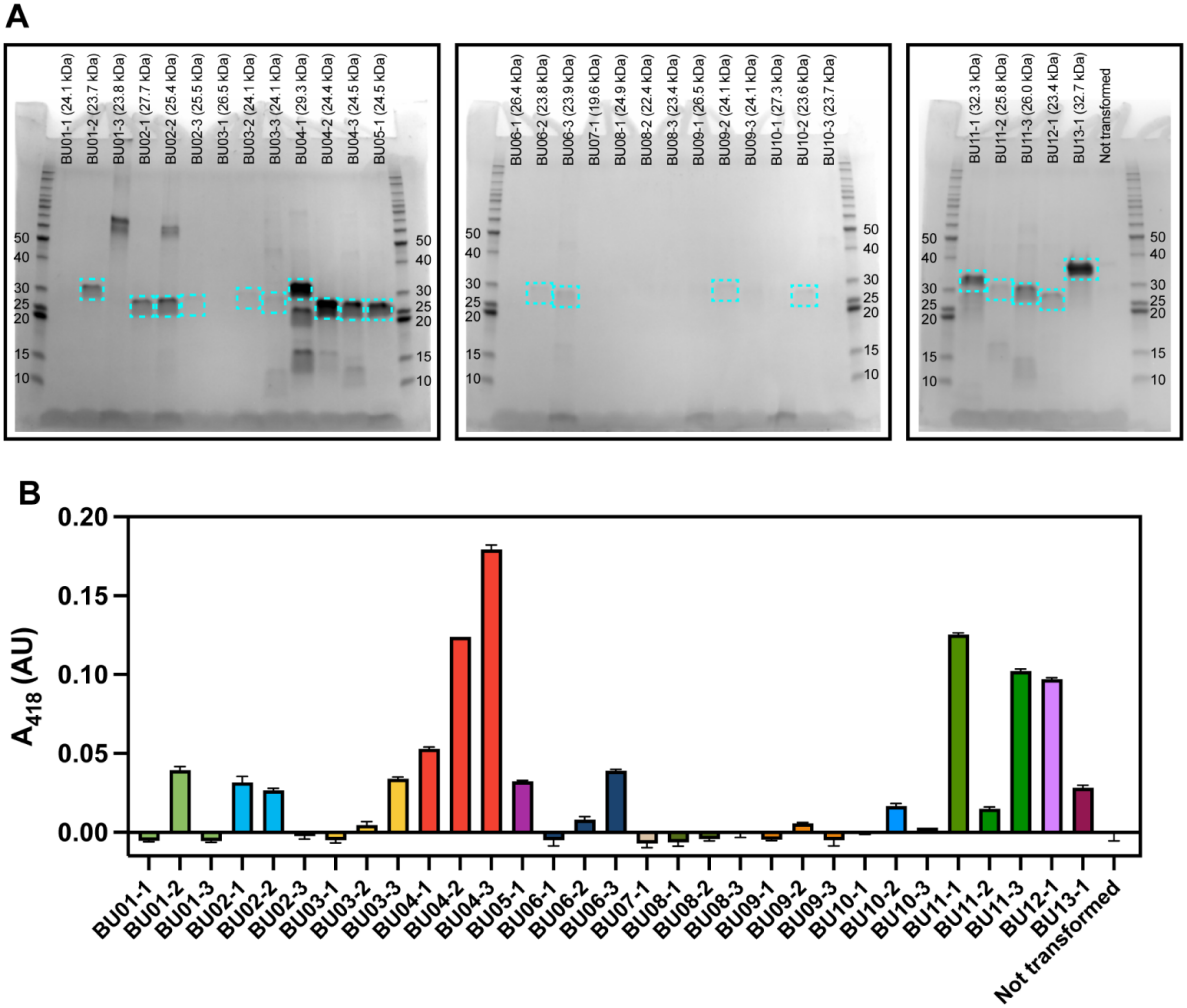
Expression and peroxidase activity of 13 putative bacterial UPOs. Panel **A** shows SDS-PAGE analysis of partially purified putative BUPOs produced recombinantly in *E. coli* (see Table S2 for details of the plasmids used). The gel was stained with Imperial Protein Stain (Thermo Fisher Scientific). The sizes of the protein MW standard (Benchmark protein ladder, Thermo Fisher Scientific) are indicated in kDa next to the corresponding bands, and the expected size of each protein is indicated in parentheses on top of each lane. Observable bands identified as BUPOs are enclosed in cyan boxes; other bands are considered contaminants. Panel **B** shows the peroxidase activity in the partially purified protein solutions. Here, 10 μL of each protein solution was used in a peroxidase activity assay with 0.8 mM ABTS and 1 mM H_2_O_2_ in 50 mM sodium phosphate buffer, pH 6.0, in a final volume of 100 μL. The reactions were incubated for 1 h at room temperature, and product formation was determined by measuring absorbance at 418 nm. The absorbance values measured immediately after starting the reactions were subtracted from the endpoint measurements to account for background signals.

Guided by expression levels and diversity criteria (i.e., including representatives from each of the most distinct clades), three enzymes were selected for further characterization: *Age*BUPO (BU04–1) from *Archangium gephyra* (clade III, UniProt accession A0A2W5 V8F7), *Kal*BUPO (BU06–2) from *Ketobacter alkanivorans* (clade I, A0A2K9LIB1), and *Hyd*BUPO (BU12–1) from *Hydrogenophaga* *sp*. A37 (clade V, A0A1 V3RV63). All three proteins were purified to homogeneity (Figure S6) yielding 40 mg, 10 mg, and 10 mg of heme-loaded enzyme per liter of culture for *Age*BUPO, *Kal*BUPO and *Hyd*BUPO, respectively, based on absorbance at 420 nm (Soret band). These yields are notable in the context of heterologous UPO production in *E. coli*. Previous reports of purified fungal UPOs from *E. coli* describe lower yields, such as 0.4–1.0 mg L^–1^ for two short fungal UPOs,^46^ whereas yields of 3–7 mg L−1 ^46^ and up to 24 mg L−1 ^47^ have been reported for partially purified preparations. Thus, while the yields obtained for *Kal*BUPO and *Hyd*BUPO fall within the broader range of previously reported *E. coli* UPO production yields, the 40 mg L^–1^ obtained for *Age*BUPO is, to the best of our knowledge, the highest reported yield for a homogeneous, heme-loaded soluble UPO purified from an *E. coli* culture.

After purification, the UV–vis spectra of the resting state, dithionite-reduced, and CO-bound proteins were measured. In the resting state, the BUPOs feature the characteristic Soret band with maxima at 423 nm for *Age*BUPO, 421 nm for *Kal*BUPO, and 417 nm for *Hyd*BUPO, as well as additional peaks at 356 nm, 537–542 nm, and 573–578 nm (Figure 4). Upon reduction with sodium dithionite, the Soret maxima shifted to 403 nm (*Age*BUPO), 408 nm (*Kal*BUPO), and 404 nm (*Hyd*BUPO), and upon CO binding, the Soret maxima shifted to 441 nm (*Age*BUPO), 443 nm (*Kal*BUPO), and 441 nm (*Hyd*BUPO), similar to what has been reported for fungal UPOs.^1,48^

**Figure 4:**
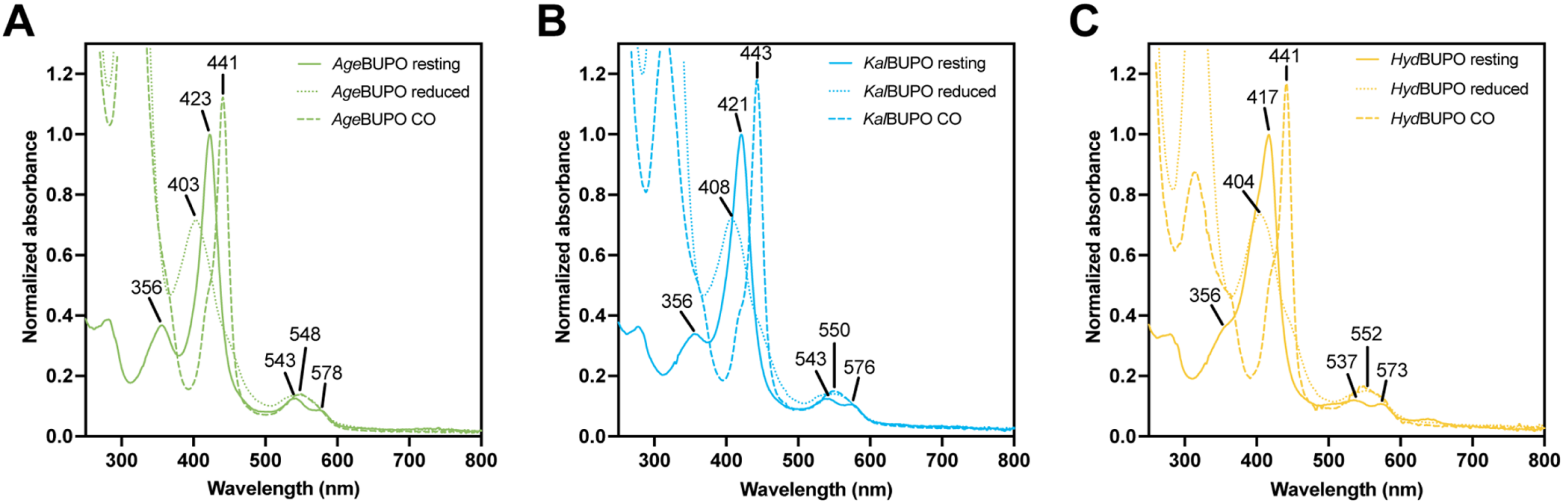
Spectrophotometric analysis of the resting state and reduced BUPOs. The spectra of (A) *Age*BUPO, (B) *Kal*BUPO, and (C) *Hyd*BUPO were recorded at approx.10 μM protein in 20 mM sodium phosphate buffer at pH 7.0, in the resting state (solid lines), after the addition of sodium dithionite (reduced; dotted lines), and in complex with CO (dashed lines). The spectra were normalized to the resting state Soret maxima.

To confirm the amounts of heme-loaded enzyme and investigate the possible presence of a magnesium-binding site in the BUPOs, the iron and magnesium contents were measured through inductively coupled plasma mass spectrometry (ICP-MS). Based on the determined iron contents, the BUPO extinction coefficients at the Soret maxima were determined to be 101.62, 106.57, and 110.05 mM^–1^ cm^–1^ for *Age*BUPO, *Kal*BUPO and *Hyd*BUPO, respectively. Interestingly, the magnesium content was negligible in all three BUPOs, whereas iron and magnesium were almost equimolar in *Hsp*UPO (Figure S7). In fungal UPOs, magnesium contributes to stabilizing the strained heme cofactor.^13^ The negligible magnesium content in BUPOs thus points to an alternative evolutionary solution for maintaining heme stability in bacterial enzymes.

### BUPOs are Capable of One- and Two-Electron Oxidations of Aromatic Substrates

Given the active site similarities observed between fungal UPOs and BUPOs, it was of interest to determine whether these bacterial proteins were capable of both one- (peroxidase) and two-electron (peroxygenase) oxidations of typical chromogenic UPO substrates. Indeed, the rapid functional screen had already indicated peroxidase activity toward ABTS (Figure 3). Using the same substrate, pH activity profiles were determined for three BUPOs, revealing a pH optimum of 7.0 for *Hyd*BUPO and 5.6 and 5.0 for *Age*BUPO and *Kal*BUPO, respectively (Figure 5). *Hsp*UPO exhibited an optimum around pH 5.0 and also showed substantially higher peroxidation rates of ABTS than those of the BUPOs.

**Figure 5:**
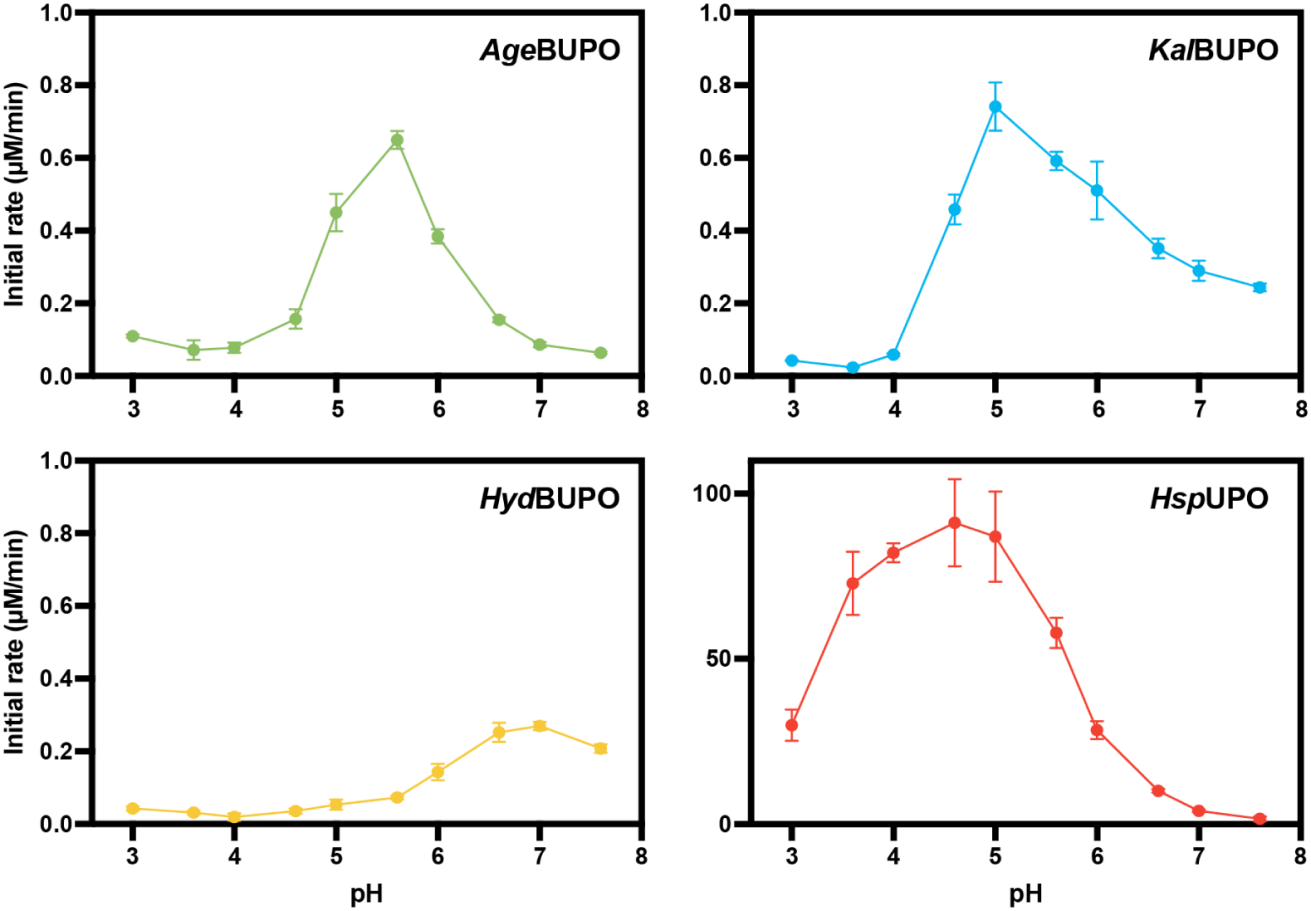
pH dependence of peroxidase activity by BUPOs and *Hsp*UPO. Peroxidase activity was determined using an ABTS time-course assay, and initial rates were obtained from the linear portion of the progress curve. The reactions were carried out in McIlvaine (∼75 mM) buffer at pH values ranging from 3.0 to 7.6, using 0.1 μM enzyme, 0.8 mM ABTS, and 1 mM H_2_O_2_ in a total volume of 200 μL. The error bars indicate the standard deviation of replicates (*n* = 3).

Peroxidase activity of the three selected purified BUPOs was further explored using 2,6-dimethoxyphenol (2,6-DMP) as a substrate, which dimerizes to produce coerulignone after one-electron oxidation.^43^ ABTS oxidation was included for comparison, with hemin and FeCl_3_ added as controls due to the low activity observed for *Hyd*BUPO toward this substrate (Figure 5). All BUPOs showed peroxidase activity with at least one of the substrates, with considerable variation among the enzymes (Figure 6 and Table 1). *Age*BUPO and *Kal*BUPO showed initial rates of 10.4 and 11.8 min^–1^, respectively, in reactions with 0.8 mM ABTS at pH 5.5 (Figure 6). *Hyd*BUPO, on the other hand, showed little activity toward this substrate under these conditions, exhibiting only a marginally higher rate compared to hemin chloride at the same concentration (0.5 μM). Under these conditions, the fungal enzyme *Hsp*UPO, used as a positive control, was approximately 40 times faster (428 min^–1^). In the 2,6-DMP assay, carried out at pH 6.0, *Kal*BUPO showed the highest rate among the BUPOs (15.2 min^–1^), followed by *Hyd*BUPO (9.0 min^–1^), while *Age*BUPO reached only 2.6 min^–1^ (Figure 6 and Table 1). Again, *Hsp*UPO showed substantially higher activity than the BUPOs (1506 min^–1^).

**Table 1:** Initial Rates for the Oxidation of Five Model Substrates by *Age*BUPO, *Kal*BUPO, *Hyd*BUPO, and *Hsp*UPO^a^

Enzyme	ABTS (min^–1^)	2,6-DMP (min^–1^)	Indole (min^–1^)	Naphthalene (min^–1^)	Veratryl alcohol (min^–1^)
*Age*BUPO	10.4 ± 0.6	2.6 ± 0.02	0.18 ± 0.01	3.5 ± 0.1	0.3 ± 0.01
*Kal*BUPO	11.8 ± 1.4	15.2 ± 0.2	3.1 ± 0.02	1.5 ± 0.04	7.6 ± 0.08
*Hyd*BUPO	1.6 ± 0.08	9.0 ± 0.2	0.24 ± 0.2	3.3 ± 0.1	5.5 ± 0.1
*Hsp*UPO	428 ± 44	1506 ± 74	15.7 ± 0.6	60 ± 0.4	52.3 ± 0.9

a Initial Rates Were Calculated from the Early Linear Portions of the Progress Curves Shown in Figure 6, Except for KalBUPO Acting on Indole and Naphthalene, Where the Rate was Determined after the Lag Phase.

**Figure 6:**
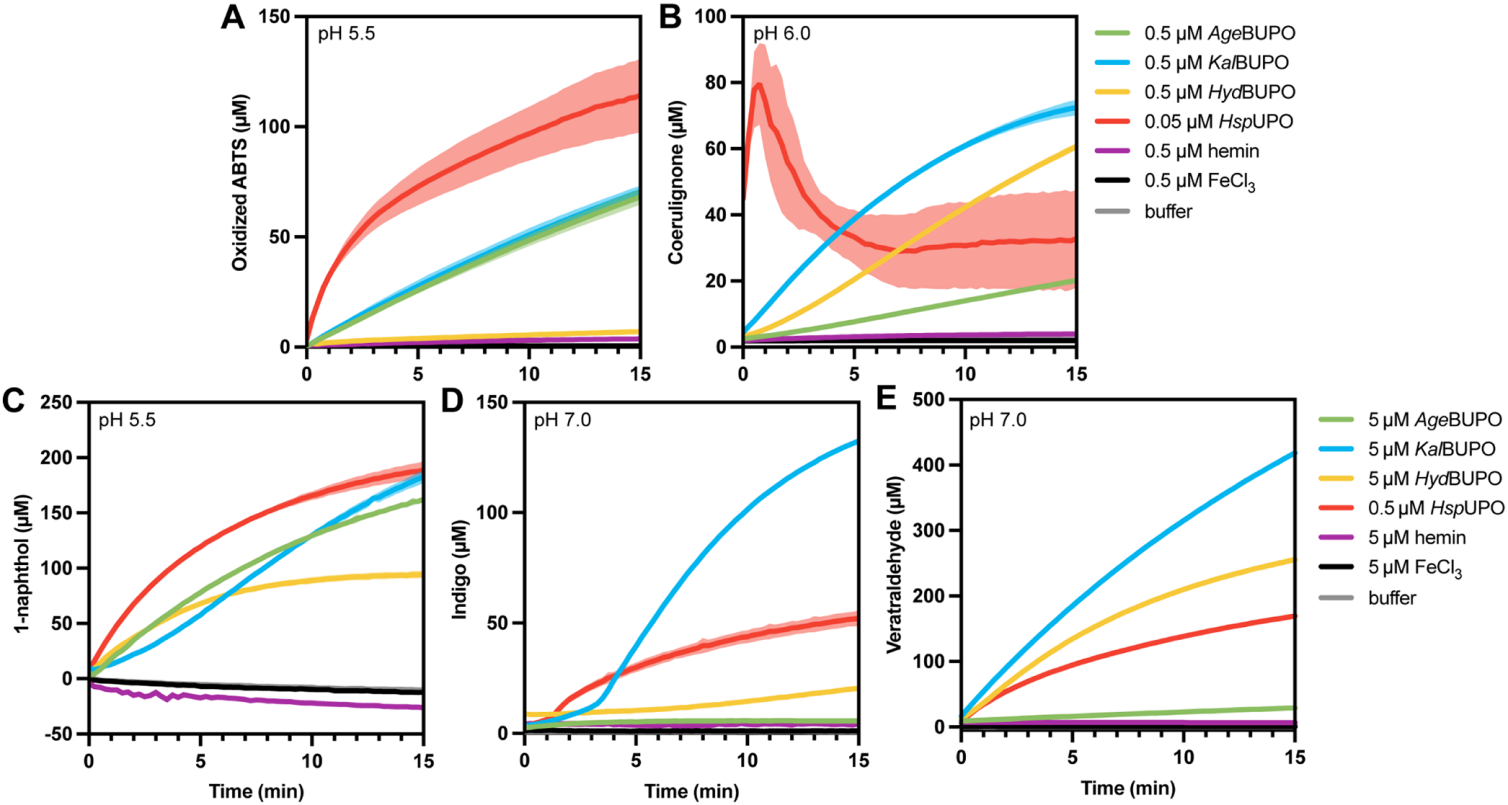
Time-course of peroxidase and peroxygenase activity by BUPOs and *Hsp*UPO. Activity was investigated using five different spectrophotometric assays: (A) ABTS, 418 nm (ε_418_ = 36 mM^–1^ cm^–1^);^43^ (B) 2,6-DMP, 469 nm (ε_469_ = 27.5 mM^–1^ cm^–1^);^43^ (C) indole, 670 nm (ε_670_ = 4.8 mM^–1^ cm^–1^);^43^ (D) naphthalene, 324 nm (quantified with a standard curve of 1-naphthol); and (E) veratryl alcohol, 310 nm (ε_310_ = 9.3 mM^–1^ cm^–1^)^1^. All reactions were measured for 15 min in 96-well plates, using 0.5 μM enzyme, hemin chloride, or FeCl_3_ (0.05 μM for *Hsp*UPO) for the peroxidase reactions on ABTS and 2,6-DMP, and 5 μM enzyme, hemin chloride, or FeCl_3_ (0.5 μM for *Hsp*UPO) for the peroxygenase reactions on naphthalene, indole, and veratryl alcohol. Further details on the reaction conditions are provided in the Materials and Methods section. Note that the high standard error and later signal decrease observed for this enzyme in panel B were caused by product precipitation. The error envelopes, in some cases too small and thus hidden behind the traces, indicate standard deviations between replicates (*n* = 3).

Catalyzing peroxygenase reactions is what makes UPOs interesting for producing industrially relevant molecules.^13^ Two examples of such molecules are 1-naphthol, which is a precursor for herbicides, insecticides, and pharmaceuticals,^49^ and indigo, which is a widely used dye.^43^ These two compounds are also used as colorimetric indicators of peroxygenase reactions, since naphthalene and indole can be hydroxylated by UPOs to produce 1-naphthol and indigo (upon dimerization of 3-hydroxyindole), respectively.^43^ Additionally, a commonly used substrate for the evaluation of UPO activity is veratryl alcohol, which can be converted by UPOs to veratraldehyde and subsequently to veratric acid through sequential hydroxylations of the benzylic carbon.^50^ All three bacterial enzymes showed peroxygenase activity toward naphthalene at pH 5.5, with initial rates (ranging from 1.5 to 3.5 min^–1^) being about 20-fold lower compared to fungal *Hsp*UPO (Figure 6 and Table 1). Regarding the oxidation of indole, only *Kal*BUPO showed clear activity, with an initial rate (3.1 min^–1^) that was only ∼5-fold lower than the rate observed for fungal *Hsp*UPO. Finally, clear conversion of veratryl alcohol to veratraldehyde at pH 7.0 was observed for *Kal*BUPO and *Hyd*BUPO, with initial rates of 7.6 and 5.5 min^–1^, respectively, between 5- and 10-fold lower than *Hsp*UPO. The peroxidase and peroxygenase activities of fungal UPOs are known to vary substantially,^17^ and the activities observed for the BUPOs toward the model substrates used here fall within the range reported for fungal UPOs. Together, these results confirm the ability of BUPOs to catalyze both one- and two-electron oxidation reactions and thus their functional similarity to fungal UPOs.

### BUPOs Can Oxidize Aliphatic Substrates

The hallmark feature of unspecific peroxygenases is their ability to perform two-electron oxidations on a broad range of substrates. After observing activity on the aromatic substrates described above, we carried out additional experiments with 3-phenyl-1-propanol (3PP), palmitic acid, and 16-hydroxypalmitic acid to assess the capacity of BUPOs to oxidize aliphatic compounds. While 3PP is an aromatic compound, it has an aliphatic moiety that is expected to be oxidized by at least some canonical fungal UPOs.^51,52^ Indeed, a control experiment with *Hsp*UPO (Figure S8) showed enzyme-dependent formation of 3-phenylpropionic acid (3PP-A), along with other oxidation products. In comparison, *Kal*BUPO also generated considerable amounts of 3PP-A, while only trace amounts were detected for *Hyd*BUPO and none for *Age*BUPO (Figure S8). Substrate depletion experiments showed similar turnover of 3PP for *Hsp*UPO and *Kal*BUPO after 1 h of reaction, although the fungal enzyme is much faster than the bacterial enzyme (Figure 7A,B).

**Figure 7:**
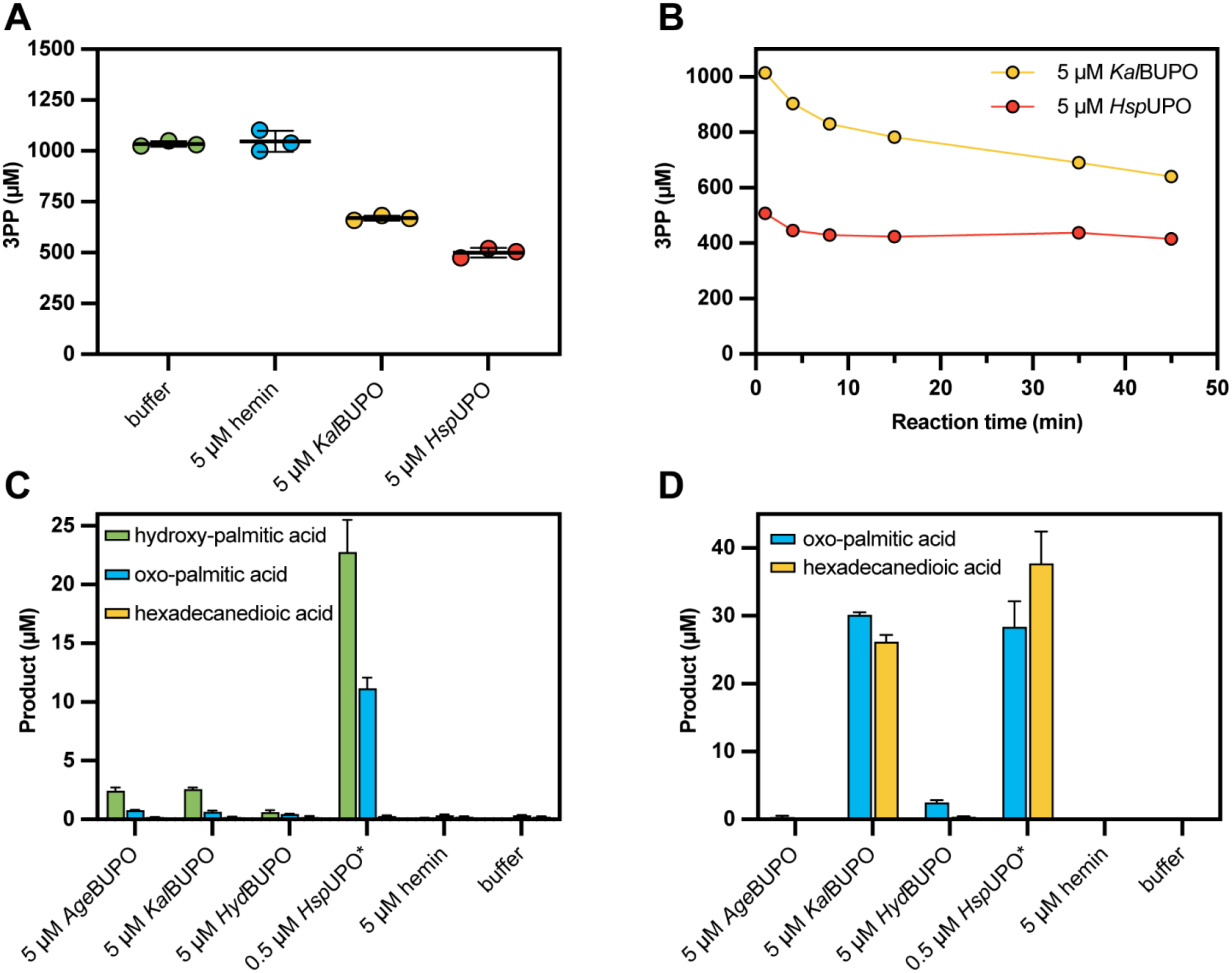
Reactions of BUPOs and *Hsp*UPO with 3-phenyl-1-propanol, palmitic acid and 16-hydroxypalmitic acid. Panel A shows substrate depletion in one-hour reactions with 3PP, while panel B shows reaction time courses for the only two enzymes for which significant 3PP-oxidizing activity was detected, *Kal*BUPO and *Hsp*UPO. The experiments shown in A and B were carried out in 50 mM sodium phosphate buffer, pH 6.0, at 30 °C (A) or at room temperature (B). For the time-course experiments (B), reactions were stopped by the addition of catalase (250 U mL^–1^). Panels C and D show the product formation in reactions with palmitic acid and 16-hydroxypalmitic acid, respectively, carried out with 5 μM BUPO or hemin chloride, or with 0.5 μM *Hsp*UPO, using 100 μM substrate and 1 mM H_2_O_2_ in 10 mM ammonium acetate, pH 6.0, and 20% acetone. The reactions were incubated for 1 h at 30 °C, and the products were quantified through LC-MS, using the extracted ion chromatograms of the [M-H]^−^ *m*/*z* values expected from the hydroxylated products (271.23), aldehyde/ketones (269.21, labeled oxo-palmitic acid), and hydroxy-ketone or dicarboxylic acid (285.21; labeled hexadecanedioic acid). Error bars in panels A, C, and D indicate standard deviations between triplicates, while horizontal lines in A denote mean values. Each point in panel B is a single measurement. Note that most of the substrate depletion by *Hsp*UPO happened before the first measurement and is not captured in the progress curve.

Encouraged by the activity of *Kal*BUPO toward 3PP, we further explored its activity toward purely aliphatic compounds, using palmitic acid as the substrate. In reactions with 100 μM palmitic acid and 5 μM enzyme, only 0.6–2.6 μM of hydroxylated product were detected (Figure 7C), which translates to less than 1 turnover per enzyme. Nonetheless, these product levels were significantly higher than the levels observed in the reactions with hemin chloride instead of the enzyme (0.05 ± 0.1 μM). Using the same conditions, 0.5 μM *Hsp*UPO produced 22.8 μM hydroxy and 11.2 μM ketone/aldehyde products (Figure 7C), which corresponds to approximately 90 turnovers, considering that ketone/aldehyde products are the result of two consecutive oxidations of palmitic acid.

Given that varying amounts of carbonyl products (ketone/aldehyde) were detected in reactions with palmitic acid, we decided to use the terminally hydroxylated compound 16-hydroxypalmitic acid as a substrate (100 μM; Figure 7D). For this substrate, *Kal*BUPO showed considerably higher activity, with 5 μM enzyme producing 30.2 μM of aldehyde (one two-electron oxidation) and 26.8 μM of hexadecanedioic acid (two two-electron oxidations), which corresponds to 57% conversion of the substrate and 17 turnovers per enzyme. Similarly, *Hsp*UPO (0.5 μM) was more active on the hydroxylated substrate, producing 28.4 μM of aldehyde and 37.7 μM of hexadecanedioic acid, which corresponds to 66% substrate conversion and 208 turnovers per enzyme. On the other hand, *Age*BUPO showed negligible activity on this substrate, while *Hyd*BUPO produced a total of 2.9 μM products, meaning less than one turnover per enzyme.

Taken together, the results from the 3PP and 16-hydroxypalmitic acid assays indicate that *Kal*BUPO and, to a lesser extent, *Hyd*BUPO can oxidize primary alcohol groups in both aromatic and aliphatic substrates, while marginal activity of the three BUPOs was detected toward unactivated aliphatic C–H bonds in palmitic acid.

## Discussion

Since their discovery, UPOs have been considered restricted to eukaryotes, largely dominated by fungi, because even sensitive sequence-profile searches failed to detect counterparts in prokaryotes.^13^ Exploiting the greater evolutionary conservation of 3D structure over primary sequence, we performed structure-based searches seeded with a fungal UPO and identified previously unrecognized bacterial proteins that are distantly related to fungal UPOs. Despite their low sequence identity, the structural similarity between bacterial and fungal UPOs, together with conserved active-site features and experimentally demonstrated H_2_O_2_-dependent oxyfunctionalization activity, supports their classification as remote homologues. This interpretation follows the Structural Classification of Proteins (SCOP) framework, in which proteins with low sequence identity may still be placed in the same superfamily when they share the same fold and functional features, implying probable common ancestry.^53,54^ In this sense, BUPOs are best regarded as a distant bacterial branch of the heme-thiolate peroxidase superfamily,^55^ also termed the peroxidase-peroxygenase superfamily.^56^

Before this work, a 2024 patent application and related EP, CN, and JP filings used the term “bacterial unspecific peroxygenases (BUPO’s)” to describe two groups of bacterial enzymes with reported peroxygenase activity toward diverse substrates, with particular emphasis on melanin production in bacterial cell factories.^57^ One of these enzyme groups is related to the 3-methyltyrosine hydroxylase SfmD from *Streptomyces lavendulae* ^58^ while the other is related to the tyrosine hydroxylase Orf13/LmbB2 from *Streptomyces refuineus*.^59^ These enzymes are histidine-ligated heme proteins and are not structurally or sequentially related to fungal UPOs. Another group of bacterial enzymes with peroxygenase activity is the widely studied cytochrome P450 family CYP152, also known as fatty acid peroxygenases (EC 1.11.2.4), which includes P450_BSβ_ from *Bacillus subtilis* ^60^ and OleT_JE_ from *Jeotgalicoccus* sp.^61^ However, although P450s share the proximal cysteine coordination of the heme iron with UPOs, they are structurally unrelated to UPOs, lack the PCP motif, and do not contain the distal acid–base pair characteristic of UPOs.^13^ Instead, in fatty acid peroxygenases, the carboxylate group of the fatty acid substrate, together with a conserved arginine residue, mediates H_2_O_2_ activation.^62^ Therefore, the BUPOs described in this study represent, to our knowledge, the first reported bacterial heme-thiolate peroxygenases that combine experimentally demonstrated peroxygenase activity with a clear structural relationship to the widely studied fungal UPOs.

The hallmark of UPO catalysis is H_2_O_2_-driven oxyfunctionalization, i.e., insertion of oxygen into C–H/C=C bonds, alongside one-electron (peroxidase-type) oxidations across diverse substrates. The three BUPOs characterized in this work fulfill these criteria: they oxidize canonical UPO substrates (e.g., ABTS and naphthalene) as well as other compounds (e.g., 3-phenyl-1-propanol, palmitic acid, and 16-hydroxypalmitic acid), using H_2_O_2_ as the cosubstrate. Compared to the reference fungal UPO, the apparent reaction rates of the BUPOs are generally lower. Whether this reflects intrinsic catalytic differences or suboptimal assay conditions remains unclear, as the biologically relevant substrates of BUPOs, and of many fungal UPOs, remain unknown. Importantly, reported peroxidase and peroxygenase activities of fungal UPOs span several orders of magnitude, and the activities observed here are consistent with this broad range.

An intriguing difference that separates the BUPOs from fungal UPOs is the presence of a second access channel to the heme, which exposes one of the heme propionate groups to solvent. In fungal UPOs, this same propionate group participates in coordinating the Mg^2+^ ion that helps stabilize the strained porphyrin system,^13^ suggesting that the bacterial enzymes may have evolved a distinct structural solution with potential functional implications. Comparable multitunnel architectures are observed in other heme enzymes, including dye-decolorizing peroxidases (DyPs) and cytochrome P450s. In DyPs, two channels lead to opposite sides of the heme, where the largest tunnel of the two exposes a propionate to solvent,^63^ resembling the secondary channel identified in BUPOs. Although the role of this channel in DyPs remains uncertain, it has been proposed to accommodate bulky aromatic substrates, such as monolignols, for oxidation.^63^ In cytochrome P450s, multiple dynamic channels have been described, providing pathways for substrates, cosubstrates, water, and protons.^64^ By analogy, the presence of two channels in BUPOs may enable complementary functions, one serving as an entry route for the cosubstrate and the other facilitating electron transfer from the main substrate to the heme. This architectural divergence from fungal UPOs may therefore reflect a bacterial adaptation toward broader substrate access or different dynamics of (co)­substrate flowing in and out of the active site.

Given the pronounced structural differences between BUPOs and fungal UPOs, it is not surprising that their reactivity profiles diverge. The BUPOs characterized here catalyze the same types of peroxygenase reactions as fungal UPOs but generally with lower efficiency. Nonetheless, *Hsp*UPO, used as a reference, is among the most efficient members of its family^65^ on many typical UPO substrates, which may accentuate the contrast. Exceptions, such as *Kal*BUPO, which shows marked activity toward 16-hydroxypalmitic acid, suggest that the natural substrates of these bacterial enzymes differ substantially from the model compounds used here.

While fungal UPOs are highly efficient biocatalysts, their heterologous production remains challenging, often requiring extensive screening and optimization.^16^ In contrast, bacterial enzymes are typically more accessible for recombinant production and engineering, and our expression screen showed that 10 of 13 BUPO genes yielded active enzymes without any optimization. The three BUPOs characterized here were produced in a standard *E. coli* strain using only codon optimization and His-tag addition, yielding 10–40 mg L^–1^ of homogeneous, heme-loaded enzyme. Although the yields obtained for *Kal*BUPO and *Hyd*BUPO are comparable to previously reported semipurified or partially purified UPO preparations from *E. coli*, the 40 mg L^–1^ obtained for *Age*BUPO appears to exceed previous reports for purified, heme-loaded, soluble UPOs from this host. Thus, the main practical advantage of BUPOs lies not only in absolute production yield but also in the speed and accessibility of bacterial expression systems for future engineering campaigns.

The physiological roles of the BUPOs remain uncertain. Their phylogenetic and structural diversity, and broad but weak activity, suggest a range of potential functions. In fungi, UPOs have been suggested to participate in processes such as xenobiotic degradation,^15^ which is also a plausible role for BUPOs. The relatively low apparent rate of BUPOs with H_2_O_2_ as a cosubstrate may indicate that these enzymes prefer alternative cosubstrates, such as organic peroxides. Thus, the reactions characterized here could represent side activities, while their main biological functions remain to be discovered. Notably, even for fungal UPOs, two decades of research have not fully clarified their native roles.

Sequence analysis revealed a relatively small BUPO family, with about 190 sequences compared to over 4 000 fungal UPOs. Despite their limited number, BUPOs are divided into highly distinct clades. Clade I BUPOs, which include *Kal*BUPO, originate from marine bacteria such as *Ketobacter alkanivorans*, an alkane-degrading species harboring *alkB* hydroxylase genes.^66^ The ability of *Kal*BUPO to oxidize aliphatic substrates such as palmitic and 16-hydroxypalmitic acids supports a potential role in alkane metabolism, possibly following initial hydroxylation by AlkB.

On a side note, nearly 70% of all identified BUPO sequences cluster in clade VI, which is found almost exclusively in *Bradyrhizobium* species. Many of these encode a bimodular protein containing both a BUPO and a diheme cytochrome c peroxidase (CCP) domain, separated by a transmembrane helix. Bacterial CCPs are known to be ubiquitous proteins whose proposed function is H_2_O_2_ detoxification^67^ and, in some cases, allowing respiration with H_2_O_2_ as the terminal electron acceptor;^68^ however, a bimodular architecture where the two modules are tethered by a transmembrane linker has never been reported before.

In summary, BUPOs represent a new family of bacterial heme-thiolate enzymes that share the structural fold and motifs of fungal UPOs. Beyond expanding the known catalytic repertoire of bacteria, these findings highlight the power of large-scale structural prediction and genome mining to uncover overlooked enzyme families. Future work should clarify the biological roles of BUPOs, explore their catalytic scope, and harness their bacterial expression advantages for enzyme engineering.

## Supplementary Material







## Data Availability

The Whole Genome Shotgun project has been deposited at DDBJ/ENA/GenBank under the accession JBRKPL000000000. The version described in this paper is version JBRKPL010000000. Nanopore sequencing raw data are available as part of NCBI BioProject PRJNA1337102. The mass spectrometry proteomics data have been deposited to the ProteomeXchange Consortium via the PRIDE (Proteomics Identification Database) partner repository with the dataset identifier PXD069273.

## Notes

Sequence identity matrix of the proteins shown in Figure 1 (Figure S1); sequence logos showing key conserved residues in BUPOs and UPOs (Figure S2); AlphaFold 3 models of representative BUPOs of clades 2, 4, and 6 (Figure S3); AlphaFold 3 models colored by pLDDT score (Figure S4); alignment of previous and new versions of *Hydrogenophaga* sp. A37 genome (Figure S5); SDS-PAGE gel of purified BUPOs (Figure S6); iron and magnesium content in the purified enzymes (Figure S7); HPLC chromatograms of BUPO and HspUPO reactions with 3PP (Figure S8); reference fungal UPOs shown in Figure 1 (Table S1); and amino acid sequences of the proteins expressed in this study (Table S2) (PDF)
Supporting dataset 1: List of the putative bacterial UPOs identified through FoldSeek and UniProt BLAST (XLSX)
Supporting dataset 2: List of proteins detected in the proteomic analysis of *Hydrogenophaga* sp. A37 and the quantification values for all biological replicates­(XLSX)

## Abbreviations

UPO: unspecific peroxygenase;
BUPO: bacterial unspecific peroxygenase.

## PROTEIN NAMES AND THEIR UNIPROT ACCESSION IDs

**Protein name**: **UniProt Identifier**
BU01–1: A0A2E0HHX6
BU01–2: A0A2E0HHX6
BU01–3: A0A2E0HHX6
BU02–1: A0A4Q6C902
BU02–2: A0A4Q6C902
BU02–3: A0A4Q6C902
BU03–1: A0A160TBE6
BU03–2: A0A160TBE6
BU03–3: A0A160TBE6
BU04–1/*Age*BUPO (native): A0A2W5 V8F7
BU04–2/*Age*BUPO (pelB-SP): A0A2W5 V8F7
BU04–3/*Age*BUPO (truncated): A0A2W5 V8F7
BU05–1: A0A0R3MHD1
BU06–1/*Kal*BUPO (native): A0A2K9LIB1
BU06–2/*Kal*BUPO (pelB-SP): A0A2K9LIB1
BU06–3/*Kal*BUPO (truncated): A0A2K9LIB1
BU07–1: A0A4Q6G3Q5
BU08–1: A0A4 V2B2U3
BU08–2: A0A4 V2B2U3
BU08–3: A0A4 V2B2U3
BU09–1: M5DTC9
BU09–2: M5DTC9
BU09–3: M5DTC9
BU10–1: A0A2D5VMJ4
BU10–2: A0A2D5VMJ4
BU10–3: A0A2D5VMJ4
BU11–1: A0A7Z9PY58
BU11–2: A0A7Z9PY58
BU11–3: A0A7Z9PY58
BU12–1/*Hyd*BUPO: A0A1 V3RV63
BU13–1: A0A2 V8LIJ3
*Hsp*UPO: A0A1Y2TH07
*Hsp*UPO-StrepTagII: A0A1Y2TH07
