# Supplementary material for: Discovery of Bacterial Unspecific Peroxygenases
Source: Biochemistry. 2026 Jun 30;65(14):2283–99. doi: 10.1021/acs.biochem.6c00230 (PMC13394420; doi:10.1021/acs.biochem.6c00230)
Supplement: Supplementary file 1 [file bi6c00230_si_001.pdf]

Supporting Information for
Discovery of bacterial unspecific peroxygenases

Esteban Lopez-Tavera, Anton A. Stepnov, Nikolai S. Ersdal, Marta Barros-Reguera, Ronja Marlonsdotter Sandholm, Sabina Leanti La Rosa, Morten Sørlie, Vincent G. H. Eijsink, and Gustav Vaaje-Kolstad*.

* To whom correspondence should be addressed: gustav.vaaje-kolstad@nmbu.no

This PDF file includes:

Figures S1 to S8
Tables S1 and S2
Legends for Supporting Datasets 1 and 2
SI References

Other supporting materials for this manuscript include the following:

Supporting Datasets 1 and 2 (Excel files)

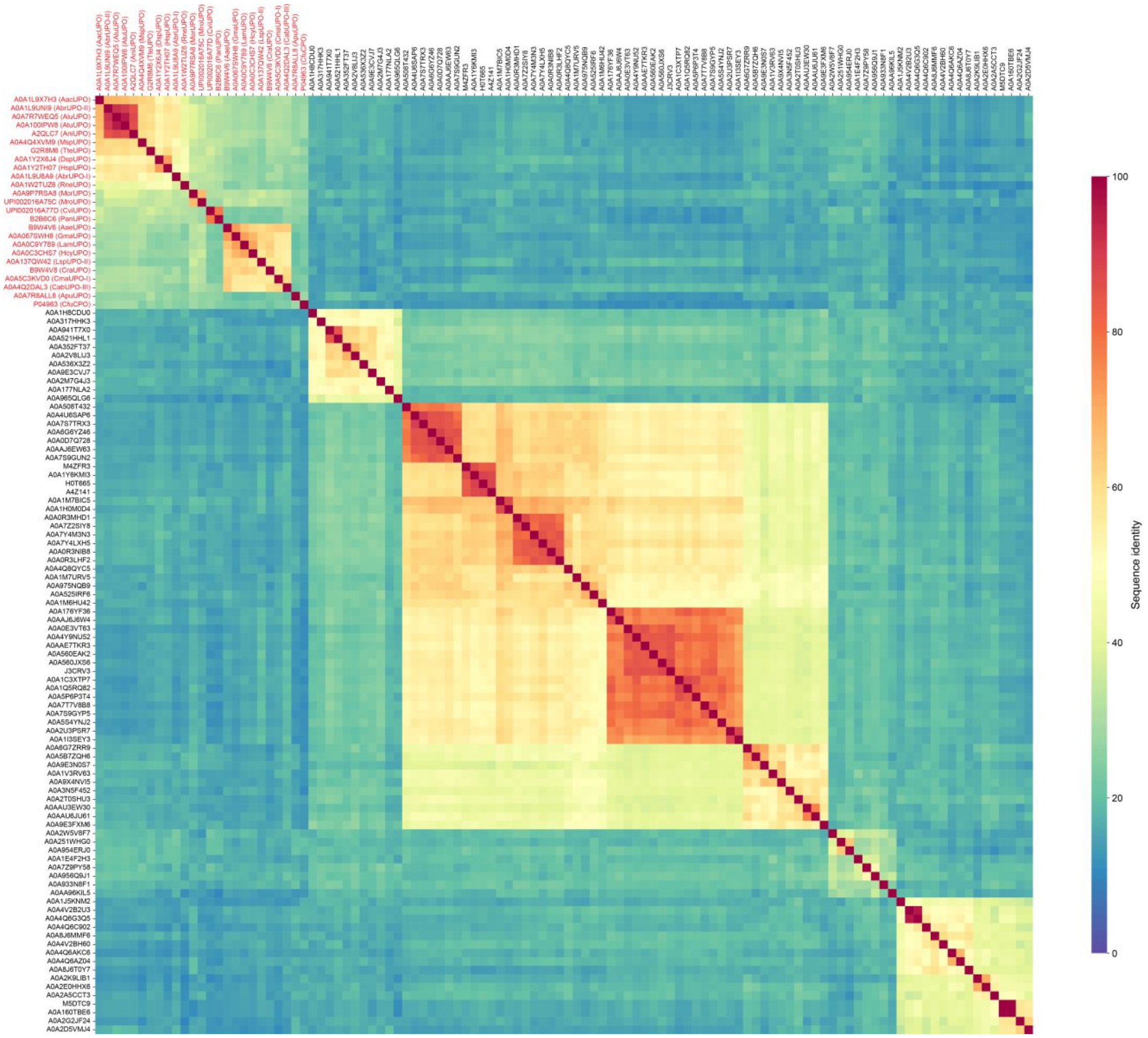

Figure S1. Sequence identity matrix of fungal UPOs and putative bacterial UPOs. The sequence identity for each protein pair is color coded according to the scale shown next to the heatmap. Fungal protein identifiers are shown as red colored text. The matrix was calculated from the multiple sequence alignment, where additional C-terminal domains, if present, were removed from the BUPO sequences before the alignment was made. The percent sequence identity (SID) was calculated as %SID = IAP × L^{-1} × 100, where IAP is the number of identical aligned non-gap residue pairs, and L is the length of the shortest protein of the pair.

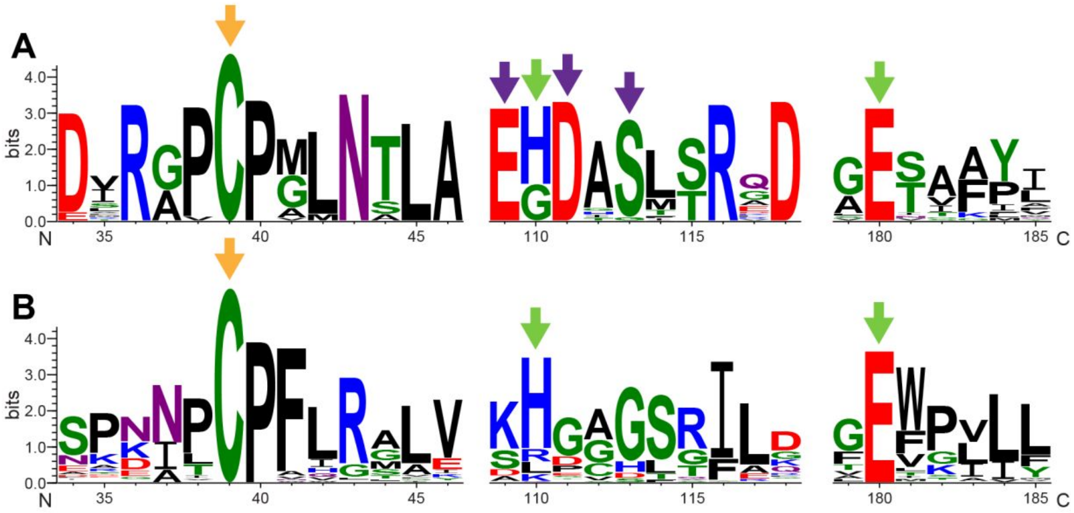

Figure S2. Sequence logos of conserved motifs in UPOs. Panel **A** shows key conserved features in fungal UPOs; specific (putative) functions are indicated with arrows as follows: **orange**, heme-coordinating cysteine; **purple**, Mg^{2+}-coordinating residues; **lime green**, acid-base catalytic pair (position His110 is exclusive to short UPOs; this base is replaced by an arginine in long UPOs, located in a region that is not shown in this figure). Panel **B** shows the equivalent positions in putative bacterial UPOs, which have the heme-coordinating cysteine and the acid-base catalytic pair, but lack the Mg^{2+}-coordinating residues. The positions shown on the horizontal axis relate to the sequence of *Hsp*UPO, including its secretion signal peptide. All logos were generated using WebLogo 3 [1] from a multiple sequence alignment made with 25 fungal and 85 bacterial sequences.

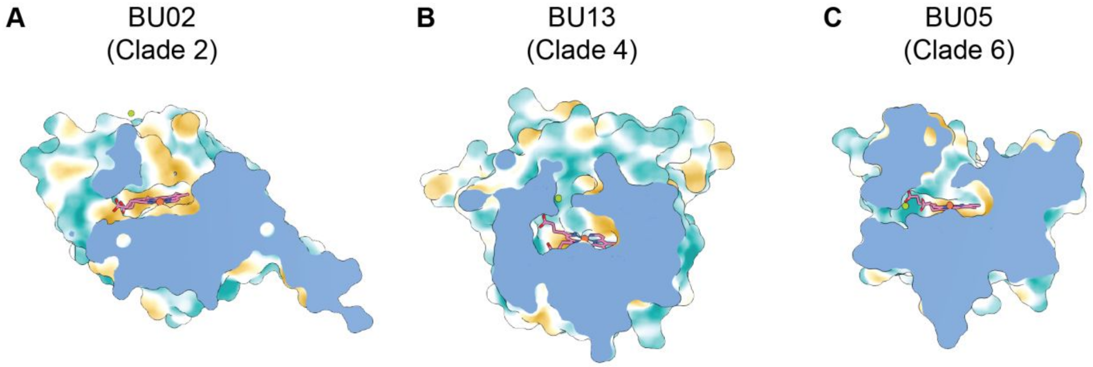

Figure S3. Active site access tunnels in BUPOs from clades 2, 4, and 6. The tunnels leading to the heme cofactor are shown by protein structure cross-sections for representatives of the clades not shown in **Figure 2**. Panel **A** shows BU02 (UniProt accession A0A4Q6C902), **B** shows BU13 (A0A2V8LIJ3), and **C** shows BU05 (A0A0R3MHD1). Note that BU13 (**B**), a representative of clade 4, does not feature a second entrance tunnel to the active site as the rest of the clades. The solvent excluded surface is shown in a lipophilicity color scale, from gold (lipophilic) to teal (hydrophilic). The models colored by pLDDT score are shown in **Figure S4**.

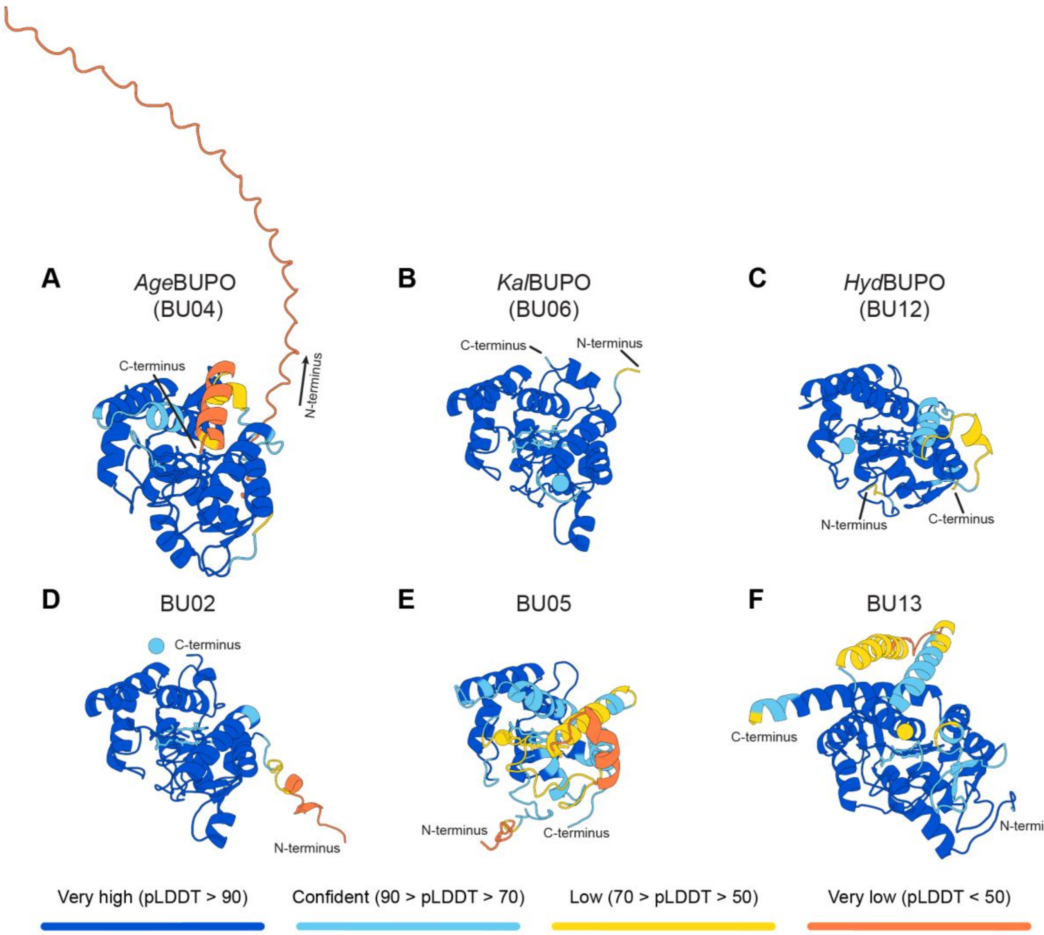

Figure S4. Structures of putative BUPOs predicted by AlphaFold 3. The structure models used in **Figures 2** and **S3** are shown colored by pLDDT values along the peptide chain and ligands (heme b cofactor and Mg^{2+} ion), using the Alphafold color scale. Panels **A-F** show the structures of *Age*BUPO (UniProt accession A0A2W5V8F7, clade 3), *Kal*BUPO (A0A2K9LIB1, clade 1), *Hyd*BUPO (A0A1V3RV63, clade 5), BU02 (A0A4Q6C902, clade 2), BU05 (A0A0R3MHD1, clade 6), and BU13 (A0A2V8LIJ3, clade 4), respectively.

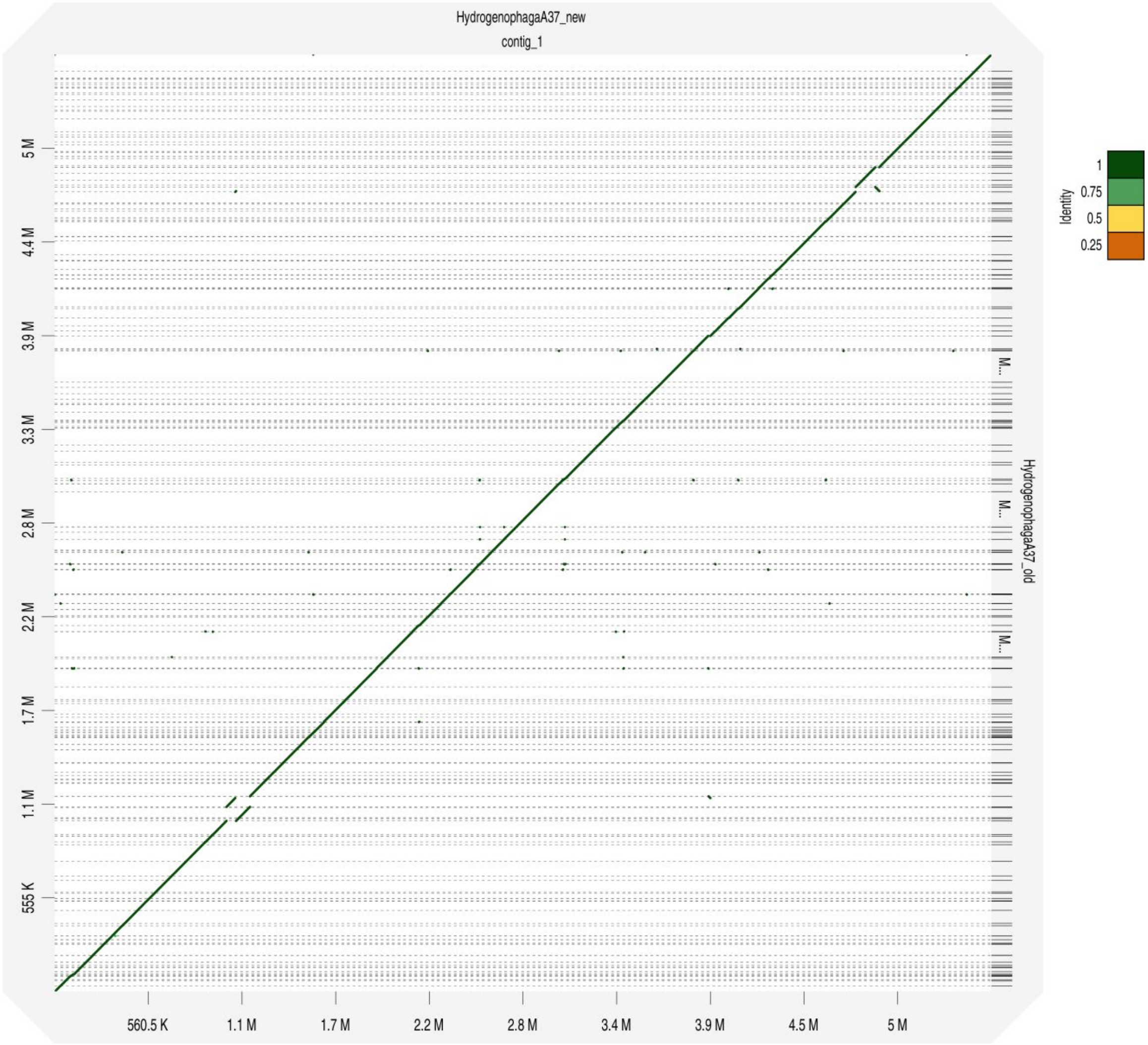

Figure S5. Alignment between the previous and new version of the genome of *Hydrogenophaga sp.* A37. The new version of the genome (GCA_053010425.1) is shown on top, while the previous version (GCA_002001205.1) is shown on the right. The alignment was visualized with D-GENIES.

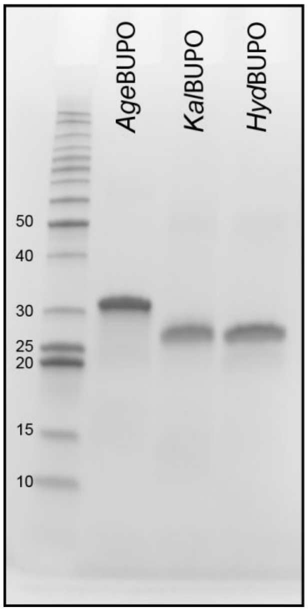

Figure S6. SDS-PAGE analysis of purified BUPOs. The gel image shows the analysis of ~1 µg purified enzyme. The relevant sizes of the protein MW standard (Benchmark protein ladder, Thermo Fisher Scientific) are indicated in kDa next to the corresponding bands. The expected sizes for *Age*UPO, *Kal*BUPO and *Hyd*BUPO are 29.3 kDa, 23.8 kDa and 23.4 kDa, respectively, without considering the mass of heme. The gel was stained with Imperial Protein Stain (Thermo Fisher Scientific).

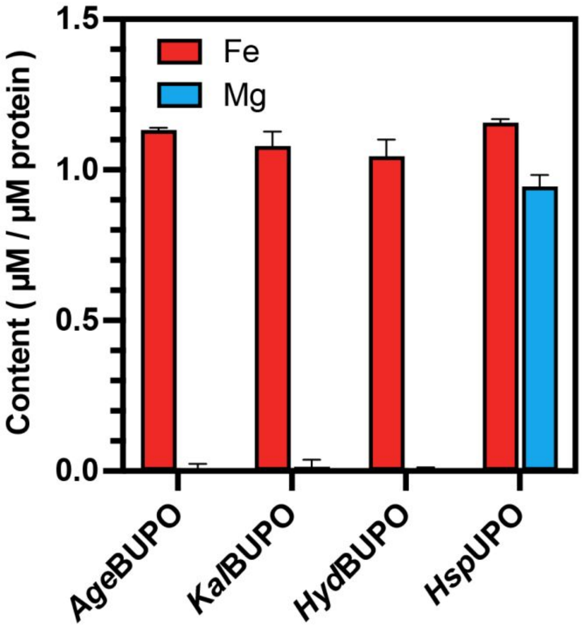

Figure S7. Iron and magnesium content in purified bacterial and fungal UPOs. The values indicate the iron and magnesium concentrations in samples of pure protein measured by ICP-MS. The values were calculated by first subtracting the background iron/magnesium content measured in the buffer, followed by division by the concentration of heme-containing protein. The latter was estimated through the absorbance at 420 nm using the extinction coefficient of *Mro*UPO ($\mathcal{E}_{420}$ = 115 mM^{-1} cm^{-1}) [2]. The error bars indicate the standard deviation of the sample measurements (n=3), including the propagation of the standard deviation from the background measurements (n=3), SD = $(SD_1^2 + SD_2^2)^{1/2}$, and divided by the concentration of heme-containing protein. The *Hsp*UPO preparation used in this analysis was produced with the affinity tag StrepTagII to ensure high purity.

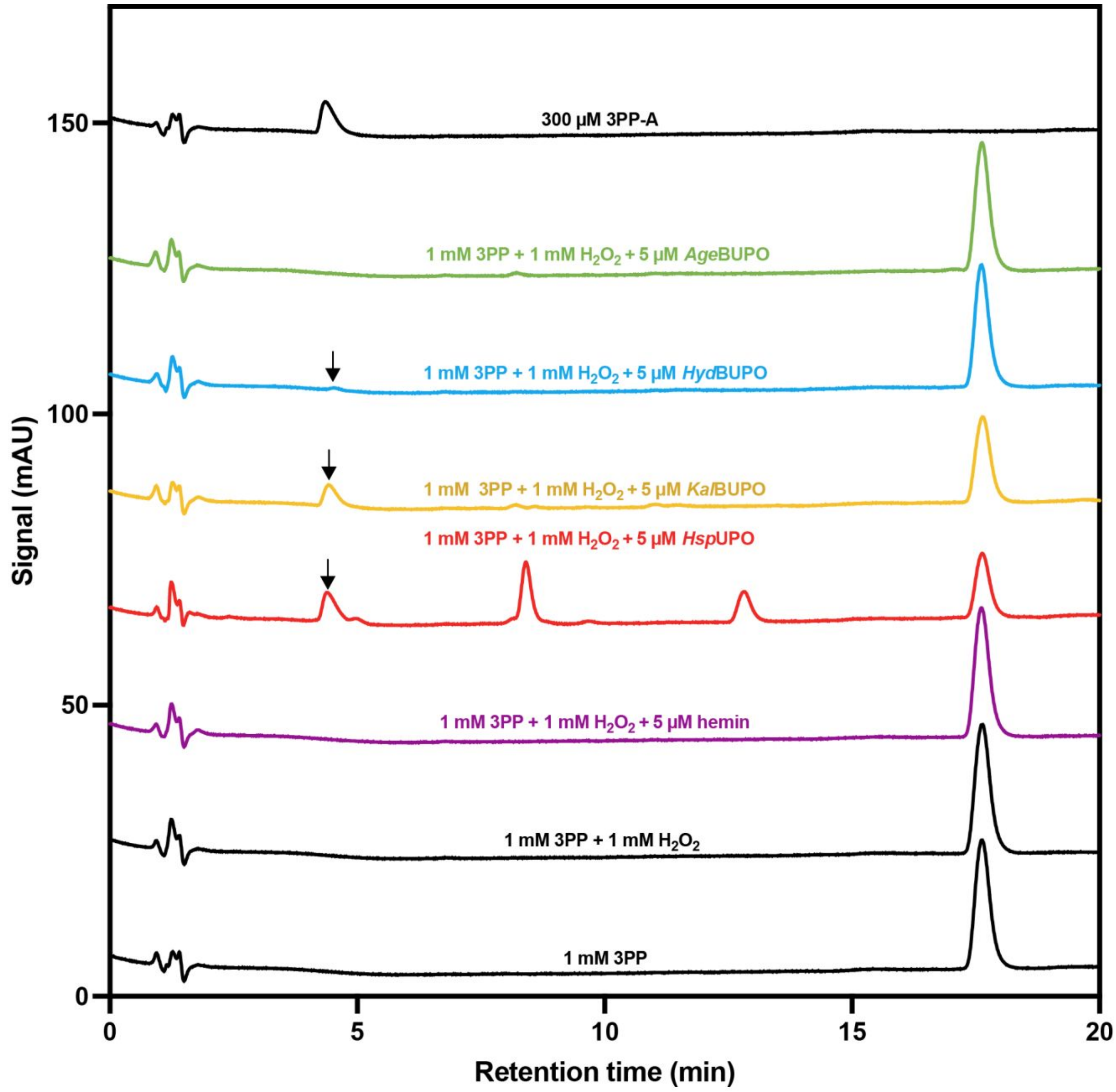

Figure S8. Enzymatic oxidation of 3-phenylpropanol (3-PP) by BUPOs and *Hsp*UPO monitored by HPLC. The chromatograms show the reaction product 3-phenylpropanoic acid (3PP-A), indicated by arrows, eluting at approx. 4.5 min, whereas the substrate elutes at approx. 18 minutes. The peaks eluting between 4.5 and 18 min are other oxidized variants of 3PP (molecular identity not determined). Standards for 3PP and 3PP-A are shown at the lower and upper chromatograms, respectively. Control reactions with either buffer or hemin are shown in chromatograms two and three, counting from the bottom, respectively. The experiments were carried out in 50 mM sodium phosphate buffer, pH 6.0, at 30 °C for 1 hour.

Table S1. Fungal UPOs used in the phylogenetic analysis. The names of the two proteins used to first detect putative BUPOs appear **bold**.

UniProt Accession	Name	Organism
A0A1L9X7H3	*Aac*UPO	*Aspergillus aculeatus*
B9W4V6	*Aae*UPO	*Agrocybe aegerita (Cyclocybe aegerita)*
A0A1L9U8A9	*Abr*UPO-I	*Aspergillus brasiliensis*
A0A1L9UNI9	*Abr*UPO-II	*Aspergillus brasiliensis*
A0A7R7WEQ5	*Alu*UPO	*Aspergillus kawachii*
A2QLC7	*Ani*UPO	*Aspergillus niger*
A0A7R8ALL8	*Apu*UPO	*Aspergillus puulaauensis*
A0A100IPW8	*Atu*UPO	*Aspergillus niger*
A0A4Q2DAL3	*Cab*UPO-III	*Candolleomyces aberdarensis*
P04963	*Cfu*CPO	*Caldariomyces fumago (Leptoxyphium fumago)*
A0A5C3KVD0	*Cma*UPO-I	*Coprinopsis marcescibilis*
B9W4V8	*Cra*UPO	*Coprinellus radians*
UPI002016A77D	*Cvi*UPO	*Collariella virescens (Achaetomiella virescens)*
A0A1Y2X6J4	*Dsp*UPO	*Daldinia sp. EC12*
A0A067SWH8	*Gma*UPO	*Galerina marginata*
A0A0C3CHS7	*Hcy*UPO	*Hebeloma cylindrosporum h7*
A0A1Y2TH07	***Hsp*UPO**	*Hypoxylon sp. EC38*
A0A0C9Y789	*Lam*UPO	*Laccaria amethystina LaAM-08-1*
A0A137QW42	*Lsp*UPO-II	*Leucoagaricus sp. SymC.cos*
A0A9P7RSA8	*Mor*UPO	*Marasmius oreades*
UPI002016A75C	***Mro*UPO**	*Marasmius rotula*
A0A4Q4XVM9	*Msp*UPO	*Monosporascus sp. 5C6A*
B2B6C6	*Pan*UPO	*Podospora anserina (Pleurage anserina)*
A0A1W2TUZ6	*Rne*UPO	*Rosellinia necatrix*
G2R8M6	*Tte*UPO	*Thielavia terrestris*

Table S2. Amino acid sequences of the proteins expressed in this study.

Given name[a]	Code name (Figure S6)	UniProt accession	Organism	Amino acid sequence[b]	SignalP prediction[c]	Supplier
	BU01-1	A0A2E0HHX6	*Pseudomonadales* bacterium	MSACGDASGPTREQLVAEFPTVERGSTRPENPEILCPFVRMLERSGLLDQTLAEQETLEVSTTELTAAADVFGCAPLECGTVAATVAVGQPGAAGVDIGRLHQAAGIAHDCGLTFAKGATQVTEARRQATLDRLALLADEQGRLTYPDLLEVKLATCAEEDVTITGAGRTETKLIFAYLGGVDNGYITLYDVESFLYASMPAVKTRYEVDLGLLSKVRAHHHHHH	OTHER	GenScript
	BU01-2	A0A2E0HHX6	*Pseudomonadales* bacterium	MKYLLPTAAAGLLLLAAQPAMAMKYLLPTAAAGLLLLAAQPAMAGDASGPTREQLVAEFPTVERGSTRPENPEILCPFVRMLERSGLLDQTLAEQETLEVSTTELTAAADVFGCAPLECGTVAATVAVGQPGAAGVDIGRLHQAAGIAHDCGLTFAKGATQVTEARRQATLDRLALLADEQGRLTYPDLLEVKLATCAEEDVTITGAGRTETKLIFAYLGGVDNGYITLYDVESFLYASMPAVKTRYEVDLGLLSKVRAHHHHHH	OTHER	GenScript
	BU01-3	A0A2E0HHX6	*Pseudomonadales* bacterium	MGDASGPTREQLVAEFPTVERGSTRPENPEILCPFVRMLERSGLLDQTLAEQETLEVSTTELTAAADVFGCAPLECGTVAATVAVGQPGAAGVDIGRLHQAAGIAHDCGLTFAKGATQVTEARRQATLDRLALLADEQGRLTYPDLLEVKLATCAEEDVTITGAGRTETKLIFAYLGGVDNGYITLYDVESFLYASMPAVKTRYEVDLGLLSKVRAHHHHHH	OTHER	GenScript
	BU02-1	A0A4Q6C902	Proteobacteria bacterium	MLIRSCLVALTGLSLSYYAFAAPGNPERLPSQIDPNISPAGFELSRAELVSRYSDIEEGSQQVENKKIVCPFLRMLERAGLFNPELETQSTLTVGIIKIASYAREFGCVVAGCGGVAAAVSAGQVTELASTPGKVNVEALHKALGISHECGLTFAKGGSVVDDATRDSTLAALKERADTLGRLTFDDLEAVKLSICEAQDVKISAPGRVEIGLIYTFLGGNERGFIDYDDVVRFFHAELPKTLGRPGIATHAHHHHHH	SP	GenScript
	BU02-2	A0A4Q6C902	Proteobacteria bacterium	MKYLLPTAAAGLLLLAAQPAMAMKYLLPTAAAGLLLLAAQPAMAAPGNPERLPSQIDPNISPAGFELSRAELVSRYSDIEEGSQQVENKKIVCPFLRMLERAGLFNPELETQSTLTVGIIKIASYAREFGCVVAGCGGVAAAVSAGQVTELASTPGKVNVEALHKALGISHECGLTFAKGGSVVDDATRDSTLAALKERADTLGRLTFDDLEAVKLSICEAQDVKISAPGRVEIGLIYTFLGGNERGFIDYDDVVRFFHAELPKTLGRPGIATHAHHHHHH	SP	GenScript
	BU02-3	A0A4Q6C902	Proteobacteria bacterium	MHHHHHHAPGNPERLPSQIDPNISPAGFELSRAELVSRYSDIEEGSQQVENKKIVCPFLRMLERAGLFNPELETQSTLTVGIIKIASYAREFGCVVAGCGGVAAAVSAGQVTELASTPGKVNVEALHKALGISHECGLTFAKGGSVVDDATRDSTLAALKERADTLGRLTFDDLEAVKLSICEAQDVKISAPGRVEIGLIYTFLGGNERGFIDYDDVVRFFHAELPKTLGRPGIATH	SP	GenScript
	BU03-1	A0A160TBE6	hydrothermal vent metagenome	MILLIRKLSLAAVFACSTLLVGCGGDVLSSEELVSLFPEVGPESTRAENTDILCPFQRMLKRSGLYDNAEDGEATSLKVKTGLASEAAEVFGCDKGSCGSIITLASIAQWNLGKLDLSRLHEAGSLLSHDCGLTFEFGGTTVSDSQRQFTLDRLLALANTEGQLQFDDLITVKQEICESQGVEMTVGGETEVKLIYAYLGGVERSFIDHSDVVRLLHATMPAYKTSAMVDLDLIGQVQAHHHHHH	LIPO	GenScript
	BU03-2	A0A160TBE6	hydrothermal vent metagenome	MKYLLPTAAAGLLLLAAQPAMAMKYLLPTAAAGLLLLAAQPAMAGGDVLSSEELVSLFPEVGPESTRAENTDILCPFQRMLKRSGLYDNAEDGEATSLKVKTGLASEAAEVFGCDKGSCGSIITLASIAQWNLGKLDLSRLHEAGSLLSHDCGLTFEFGGTTVSDSQRQFTLDRLLALANTEGQLQFDDLITVKQEICESQGVEMTVGGETEVKLIYAYLGGVERSFIDHSDVVRLLHATMPAYKTSAMVDLDLIGQVQAHHHHHH	LIPO	GenScript
	BU03-3	A0A160TBE6	hydrothermal vent metagenome	MHHHHHHGGDVLSSEELVSLFPEVGPESTRAENTDILCPFQRMLKRSGLYDNAEDGEATSLKVKTGLASEAAEVFGCDKGSCGSIITLASIAQWNLGKLDLSRLHEAGSLLSHDCGLTFEFGGTTVSDSQRQFTLDRLLALANTEGQLQFDDLITVKQEICESQGVEMTVGGETEVKLIYAYLGGVERSFIDHSDVVRLLHATMPAYKTSAMVDLDLIGQVQ	LIPO	GenScript
***Age*BUPO (native)**	BU04-1	A0A2W5V8F7	*Archangium gephyra*	MKIQPSIPSVAPSSTTPAERVTSQPTPAAPKVEGFQAPRAAPATDLEGTSHGYVAPNKVESPFTNEEKVQLARKIPCPALAGAFNAGMLKVAKDGTVKIPDLERTLGQLGAGGLVTKVLTSAADATDDVKGSFNLFKLNGSNLDHTGSTGIRQNGVHPERFEKLMSFSKDGQRLTAKDLADAAESFAKEDPGLRGRITQQAELTAVLKIFGRTAEDGSKYFMRDDAKSLFVDGQIPASWEPPAVPGKKVGLGEVLGGTALGLFRQLVNGQAHHHHHH	OTHER	GenScript
*Age*BUPO (pelB-SP)	BU04-2	A0A2W5V8F7	*Archangium gephyra*	MKYLLPTAAAGLLLLAAQPAMAMKYLLPTAAAGLLLLAAQPAMASHGYVAPNKVESPFTNEEKVQLARKIPCPALAGAFNAGMLKVAKDGTVKIPDLERTLGQLGAGGLVTKVLTSAADATDDVKGSFNLFKLNGSNLDHTGSTGIRQNGVHPERFEKLMSFSKDGQRLTAKDLADAAESFAKEDPGLRGRITQQAELTAVLKIFGRTAEDGSKYFMRDDAKSLFVDGQIPASWEPPAVPGKKVGLGEVLGGTALGLFRQLVNGQAHHHHHH	OTHER	GenScript
*Age*BUPO (truncated)	BU04-3	A0A2W5V8F7	*Archangium gephyra*	MHHHHHHASHGYVAPNKVESPFTNEEKVQLARKIPCPALAGAFNAGMLKVAKDGTVKIPDLERTLGQLGAGGLVTKVLTSAADATDDVKGSFNLFKLNGSNLDHTGSTGIRQNGVHPERFEKLMSFSKDGQRLTAKDLADAAESFAKEDPGLRGRITQQAELTAVLKIFGRTAEDGSKYFMRDDAKSLFVDGQIPASWEPPAVPGKKVGLGEVLGGTALGLFRQLVNGQ	OTHER	GenScript
	BU05-1	A0A0R3MHD1	*Bradyrhizobium lablabi*	MSDHPVATAPGTALAGQFPVSPNNPCPFLRALVANGYVGGDVVPLSQISEIVGDASGQTGLGKMKVRIATWMVAVIANGLGPGRLFKSATSGAVLDQLRDGPLDKHGGGSRILDATAKVHEEQIDRLASFGKDCKDPAGGIETGLTAKEIETFMAANIKRDGDAARWYFPILMKGEWPVLLKILGKGEGEERYLSVAEVRTLFVERRLPKRIADRLPKPASPAHHHHHH	OTHER	GenScript
*Kal*BUPO (native)	BU06-1	A0A2K9LIB1	*Ketobacter alkanivorans*	MIIIKKSTWSAALLLVATNIFVAGCSDAPGPSREQLVAEYPSVEQGSTRQENIEIMCPFVRMLERSGLFDETFANQGDLDISTSELTSAAQEFGCAVLECGTVAATAAVGQPGGSGVDIERLHEAAGIAHDCGLTFEYGGTQVSDDRRDATITRLGELADEQGHLVYDDILQVKLETCDGEGVGITTAGRTETKLIFAYLGGVDNGFVTLDDVSSFLHAEMPSVKTRFMVDARQLGKVRAHHHHHH	LIPO	Twist Bioscience
***Kal*BUPO (pelB-SP)**	BU06-2	A0A2K9LIB1	*Ketobacter alkanivorans*	MKYLLPTAAAGLLLLAAQPAMAMKYLLPTAAAGLLLLAAQPAMASDAPGPSREQLVAEYPSVEQGSTRQENIEIMCPFVRMLERSGLFDETFANQGDLDISTSELTSAAQEFGCAVLECGTVAATAAVGQPGGSGVDIERLHEAAGIAHDCGLTFEYGGTQVSDDRRDATITRLGELADEQGHLVYDDILQVKLETCDGEGVGITTAGRTETKLIFAYLGGVDNGFVTLDDVSSFLHAEMPSVKTRFMVDARQLGKVRAHHHHHH	LIPO	Twist Bioscience
*Kal*BUPO (truncated)	BU06-3	A0A2K9LIB1	*Ketobacter alkanivorans*	MHHHHHHASDAPGPSREQLVAEYPSVEQGSTRQENIEIMCPFVRMLERSGLFDETFANQGDLDISTSELTSAAQEFGCAVLECGTVAATAAVGQPGGSGVDIERLHEAAGIAHDCGLTFEYGGTQVSDDRRDATITRLGELADEQGHLVYDDILQVKLETCDGEGVGITTAGRTETKLIFAYLGGVDNGFVTLDDVSSFLHAEMPSVKTRFMVDARQLGKVR	LIPO	Twist Bioscience
	BU07-1	A0A4Q6G3Q5	Proteobacteria bacterium	MLERAGLYNKEVGMGGRLLVSISKITTLAKQWGCAIKECGLVATAVSAGQVTNLSTNPGFANLGALHRALGVSHECGFTFAKGGSVVSDEQRATSLSRLEALADSEGRLTFDNLNTVKKQICEEQGEVNTFASQVEVKLIYSFLGGKDRGFVDYDDVVRFFHAELPKTISAPSGLAHHHHHH	OTHER	Twist Bioscience
	BU08-1	A0A4V2B2U3	Pseudomonadota bacterium	MNPFKRLSTQLLCTLLLSSSAIAAPLNRAEFAEKFPQIEEGSSKPENKAIVCPFHRMLERAGLYDKEVGMGGRLLVSIAKITTLAKQWGCAIKECGTVATAVSAGQLTNLSTKPGFANLGALHRALGVSHECGFTFAKGGSVVSDEQRATSLSRLEALADSNGRLTFDNLNTVKNQICEEQGEVNTFASQVEVKLIYSFLGGKDRGFVDYDDVVRFFHAELPKTISAPSGL	SP	Twist Bioscience

	BU08-2	A0A4V2B2U3	Pseudomonadota bacterium	MKYLLPTAAAGLLLLAAQPAMAMKYLLPTAAAGLLLLAAQPAMAAPLNRAEFAEKFPQIEEGSSKPENKAIVCPFHRMLERAGLYDKEVGMGGRLLVSIAKITTLAKQWGCAIKECGTVATAVSAGQLTNLSTKPGFANLGALHRALGVSHECGFTFAKGGSVVSDEQRATSLSRLEALADSNGRLTFDNLNTVKNQICEEQGEVNTFASQVEVKLIYSFLGGKDRGFVDYDDVVRFFHAELPKTISAPSGL	SP	Twist Bioscience
	BU08-3	A0A4V2B2U3	Pseudomonadota bacterium	MHHHHHHAPLNRAEFAEKFPQIEEGSSKPENKAIVCPFHRMLERAGLYDKEVGMGGRLLVSIAKITTLAKQWGCAIKECGTVATAVSAGQLTNLSTKPGFANLGALHRALGVSHECGFTFAKGGSVVSDEQRATSLSRLEALADSNGRLTFDNLNTVKNQICEEQGEVNTFASQVEVKLIYSFLGGKDRGFVDYDDVVRFFHAELPKTISAPSGL	SP	Twist Bioscience
	BU09-1	M5DTC9	*Thalassolituus oleivorans* MIL-1	MILLIRKLSLAAVFACSTLLVGCGGDVLSSEELVSLFPEVGPESTRAENTDILCPFQRMLKRSGLYDNAEDGEATSLKVKTGLASEAAEVFGCDKGSCGSIITLASIAQWNLGKLDLSRLHEAGSLLSHDCGLTFEFGGTSVSDSQRQFTLDRLLALANTEGQLQFDDLITVKQEICESQGVEMTVGGETEVKLIYAYLGGVERSFIDHSDVVRLLHATMPAYKTSAMVDLDLIGQVQAHHHHHH	LIPO	Twist Bioscience
	BU09-2	M5DTC9	*Thalassolituus oleivorans* MIL-1	MKYLLPTAAAGLLLLAAQPAMAMKYLLPTAAAGLLLLAAQPAMAGGDVLSSEELVSLFPEVGPESTRAENTDILCPFQRMLKRSGLYDNAEDGEATSLKVKTGLASEAAEVFGCDKGSCGSIITLASIAQWNLGKLDLSRLHEAGSLLSHDCGLTFEFGGTSVSDSQRQFTLDRLLALANTEGQLQFDDLITVKQEICESQGVEMTVGGETEVKLIYAYLGGVERSFIDHSDVVRLLHATMPAYKTSAMVDLDLIGQVQAHHHHHH	LIPO	Twist Bioscience
	BU09-3	M5DTC9	*Thalassolituus oleivorans* MIL-1	MHHHHHHGGDVLSSEELVSLFPEVGPESTRAENTDILCPFQRMLKRSGLYDNAEDGEATSLKVKTGLASEAAEVFGCDKGSCGSIITLASIAQWNLGKLDLSRLHEAGSLLSHDCGLTFEFGGTSVSDSQRQFTLDRLLALANTEGQLQFDDLITVKQEICESQGVEMTVGGETEVKLIYAYLGGVERSFIDHSDVVRLLHATMPAYKTSAMVDLDLIGQVQ	LIPO	Twist Bioscience
	BU10-1	A0A2D5VMJ4	*Pseudomonas sp.*	MTFPRLSLSSLSSGITSHFARKAAAVLTLAVGMTGCGGDTYSADELAALYPQVAAGSTTPEDAEILCPFQRMIKRSGLLDDVLADGEFEVRNRLVTEASEIFGCASGACGTFVGYASLAQGNWNTLELNRLHEAGFLSHDCGLTFELGSITVDDSRRDFTLDRLTDLAVDGTLSLDNLMQVKQEICDLEGVEMTIGGETEVKLIYAYLGGSERGYVMNSDVSRFLHATLPAYKSSEYIDFSVVSEAHHHHHH	LIPO	Twist Bioscience
	BU10-2	A0A2D5VMJ4	*Pseudomonas sp.*	MKYLLPTAAAGLLLLAAQPAMAMKYLLPTAAAGLLLLAAQPAMAGGDTYSADELAALYPQVAAGSTTPEDAEILCPFQRMIKRSGLLDDVLADGEFEVRNRLVTEASEIFGCASGACGTFVGYASLAQGNWNTLELNRLHEAGFLSHDCGLTFELGSITVDDSRRDFTLDRLTDLAVDGTLSLDNLMQVKQEICDLEGVEMTIGGETEVKLIYAYLGGSERGYVMNSDVSRFLHATLPAYKSSEYIDFSVVSEAHHHHHH	LIPO	Twist Bioscience
	BU10-3	A0A2D5VMJ4	*Pseudomonas sp.*	MHHHHHHGGDTYSADELAALYPQVAAGSTTPEDAEILCPFQRMIKRSGLLDDVLADGEFEVRNRLVTEASEIFGCASGACGTFVGYASLAQGNWNTLELNRLHEAGFLSHDCGLTFELGSITVDDSRRDFTLDRLTDLAVDGTLSLDNLMQVKQEICDLEGVEMTIGGETEVKLIYAYLGGSERGYVMNSDVSRFLHATLPAYKSSEYIDFSVVSE	LIPO	Twist Bioscience
	BU11-1	A0A7Z9PY58	bacterium SCN 62-11	MNIQTRLNTYTPAAHAMDSLPRTEQEQKPVPPSEDGKFTIDDIICPFQRVAYNEGVLKVDENGNATNLPEVLKEYAGAGWGLTKVANHAAKRLSTDGSHWQALWADSYNLQDLEGSSLDHKADTQILRGGFNQERLDKALSFSSDGERLTLEDLRRFQKSNLEEEPGRHGEIFGAAELALLVKVFGRTDGTGTKFISNKDFTTIFKDNKWPEGWEPPKAGSTNFLTVAQSVNEYFSMDDKIGEAKEAVASEAAPAKGSAKQACPFLSGQPFDLAEAAKQHSDRLEAHHHHHH	OTHER	Twist Bioscience
	BU11-2	A0A7Z9PY58	bacterium SCN 62-11	MKYLLPTAAAGLLLLAAQPAMAMKYLLPTAAAGLLLLAAQPAMADSLDPAGLSDKLDNPKAALIPCPWWRTVINEDLVKVDGDGNVTMKDLRHALKATGVTFGLREGAILGVKRVAAQLAGQATGGITGFMHVLCMDKINVLDLPKSSLMHTGDSGTLRNGFNQENLERLLSFSSDGQRITANDLADANKKQVEADPGESGRKFGIAEYSILLNIFGRKDENGQKYLTKQDLTDVFKNNEFPENWEKPKVGFINLGKSIFGMFGRQKEETKAHHHHHH	OTHER	Twist Bioscience
	BU11-3	A0A7Z9PY58	bacterium SCN 62-11	MHHHHHHADSLDPAGLSDKLDNPKAALIPCPWWRTVINEDLVKVDGDGNVTMKDLRHALKATGVTFGLREGAILGVKRVAAQLAGQATGGITGFMHVLCMDKINVLDLPKSSLMHTGDSGTLRNGFNQENLERLLSFSSDGQRITANDLADANKKQVEADPGESGRKFGIAEYSILLNIFGRKDENGQKYLTKQDLTDVFKNNEFPENWEKPKVGFINLGKSIFGMFGRQKEETK	OTHER	Twist Bioscience
***Hyd*BUPO (native)**	BU12-1	A0A1V3RV63	*Hydrogenophaga sp.* A37	MHHHHHHAPTTPRQKPVSPNNPCPFLRTLVAQGLVPDDVVPIGELTDAILKVARTGEGEPTLPAAAIRAVALAANGLGPLQLLRAGMDGVALNALRGGPLDKQGAGSGILSATATVDAEQLDRLDQFASDHVNARGRKERGLDRAALDRMMDANIERAASPRLLDRQLMDGEWPILLQVMGKEGKAGRYLSVKEVSDLFLHRRFPRRMAAALKDQRG	OTHER	Twist Bioscience
	BU13-1	A0A2V8LIJ3	Acidobacteria bacterium	MNTDPIQPTADERTSVVEKKATCPFIGSAVAQDALPIRNDANDPLAGIEDVRRLGNTGGGNLGDLLVFFASGNHAFMRGASGKLDAAVPLGFFSLDFPGSQGSHPGHSGILQGDPESLNSGRFSQADFDRLINLATDGFLKRSDVGRFIAENLIRDPKSKVLDHRTVALLAGDLVHIVESGFGFIGDLIKPNQADSHRDLEEKLTKLGGEDNLVGSSGEFGLLFAFFAHKPGSKTVAGEPALDIRDLKTMFVAKRLPEGWETWKKSRIDWVTNTTGLLISAANEYRKLKGTLRAGVAHHHHHH	OTHER	Twist Bioscience
***Hsp*UPO**[d]		A0A1Y2TH07	*Hypoxylon sp.* EC38	MRFPSIFTAVLFAASSALAAPVNTTTEDETAQIPAEAVIGYSDLEGDFDVAVLPFSASIAAKEEGVSLEKREAEAAPSPSSGWQAPGPNDVRAPCPMLNTLANHGFLPHDGKGITVNKTIDALGSALNIDANLSTLLFGFAATTNPQPNATFFDLDHLSRHNILEHDASLSRQDSYFGPADVFNEAVFNQTKSFWTGDIIDVQMAANARIVRLLTSNLTNPEYSLSDLGSAFSIGESAAYIGILGDKKSATVPKSWVEYLFENERLPYELGFKRPNDPFTTDDLGDLSTQIINAQHFPQSPGKVEKRGDTRCPYGYH	SP	Twist Bioscience
***Hsp*UPO-StrepTagII**[d]		A0A1Y2TH07	*Hypoxylon sp.* EC38	MRFPSIFTAVLFAASSALAAPVNTTTEDETAQIPAEAVIGYSDLEGDFDVAVLPFSASIAAKEEGVSLEKREAEAWSHPQFEKAPSPSSGWQAPGPNDVRAPCPMLNTLANHGFLPHDGKGITVNKTIDALGSALNIDANLSTLLFGFAATTNPQPNATFFDLDHLSRHNILEHDASLSRQDSYFGPADVFNEAVFNQTKSFWTGDIIDVQMAANARIVRLLTSNLTNPEYSLSDLGSAFSIGESAAYIGILGDKKSATVPKSWVEYLFENERLPYELGFKRPNDPFTTDDLGDLSTQIINAQHFPQSPGKVEKRGDTRCPYGYH	SP	

[a] The names in **bold** correspond to the variants expressed in 1 L scale and purified (see main text).

[b] The portions of the sequences in orange are the native signal peptide (SignalP 6.0 [3]) or disordered region (in AlphaFold models) that were excluded in the truncated variants; predicted lipidation sites (SignalP 6.0 [3]) are highlighted in yellow (**C**) and were excluded in truncated variants; the added His-tag (or StrepTagII in case of *Hsp*UPO) is shown in red; the pelB (or alpha factor in case of *Hsp*UPO) signal peptide sequence is shown in blue.

[c] Result of signal peptide prediction using SignalP 6.0 [3], indicating the presence of a Sec/SPI signal peptide (**SP**), or a Sec/SPII lipoprotein signal peptide (**LIPO**). **OTHER** indicates the absence of signal peptide.

[d] *Hsp*UPO is the fungal UPO used as reference in this study.

Supporting Dataset 1 (separate file). Complete list of the putative bacterial UPOs identified through FoldSeek and UniProt BLAST, including their UniProt and NCBI accession numbers, organism of origin, sequence length, isolation source and host (if available), SignalP prediction result (SP: Sec signal peptide (Sec/SPI); LIPO: Lipoprotein signal peptide (Sec/SPII); or OTHER: No signal peptide at all), and the clade in the phylogenetic tree they belong to.

Supporting Dataset 2 (separate file). List of proteins detected in the proteomic analysis of *Hydrogenophaga sp*. A37 and the quantification values for all biological replicates. *Hyd*BUPO corresponds to the accession number MGS5087929.1.

SI References

(1) Crooks, G. E.; Hon, G.; Chandonia, J.-M.; Brenner, S. E. WebLogo: A Sequence Logo Generator. *Genome Res.* **2004**, *14* (6), 1188–1190. https://doi.org/10.1101/gr.849004.

(2) Linde, D.; Santillana, E.; Fernández-Fueyo, E.; González-Benjumea, A.; Carro, J.; Gutiérrez, A.; Martínez, A. T.; Romero, A. Structural Characterization of Two Short Unspecific Peroxygenases: Two Different Dimeric Arrangements. *Antioxidants* **2022**, *11* (5), 891. https://doi.org/10.3390/antiox11050891.

(3) Teufel, F.; Almagro Armenteros, J. J.; Johansen, A. R.; Gíslason, M. H.; Pihl, S. I.; Tsirigos, K. D.; Winther, O.; Brunak, S.; Von Heijne, G.; Nielsen, H. SignalP 6.0 Predicts All Five Types of Signal Peptides Using Protein Language Models. *Nat Biotechnol* **2022**, *40* (7), 1023–1025. https://doi.org/10.1038/s41587-021-01156-3.
